# Ethylene negatively regulates transcript abundance of ROP-GAP rheostat-encoding genes and affects apoplastic reactive oxygen species homeostasis in epicarps of cold stored apple fruits

**DOI:** 10.1093/jxb/erv422

**Published:** 2015-10-01

**Authors:** Monica Zermiani, Elisabetta Zonin, Alberto Nonis, Maura Begheldo, Luca Ceccato, Alice Vezzaro, Barbara Baldan, Annarita Trentin, Antonio Masi, Marco Pegoraro, Livio Fadanelli, William Teale, Klaus Palme, Luigi Quintieri, Benedetto Ruperti

**Affiliations:** ^1^Department of Agronomy, Food, Natural resources, Animals and Environment (DAFNAE), University of Padova, 35020 Legnaro, Italy; ^2^Department of Biology, University of Padova, 35121 Padova, Italy; ^3^Edmund Mach Foundation, San Michele all’Adige, 38123 Trento, Italy; ^4^Institute of Biology II/Molecular Plant Physiology, Faculty of Biology, Albert-Ludwigs-University of Freiburg, D-79104 Freiburg, Germany; ^5^Centre for Biological Systems Analysis, Albert-Ludwigs-University of Freiburg, D-79104 Freiburg, Germany; ^6^Freiburg Institute for Advanced Sciences (FRIAS), Albert-Ludwigs-University of Freiburg, D-79104 Freiburg, Germany; ^7^Centre for Biological Signalling Studies (bioss), Albert-Ludwigs-University of Freiburg, D-79104 Freiburg, Germany; ^8^Freiburg Initiative for Systems Biology (FRISYS), Albert-Ludwigs-University of Freiburg, D-79104 Freiburg, Germany; ^9^Department of Pharmaceutical and Pharmacological Sciences, University of Padova, Padova, 35131 Padova, Italy

**Keywords:** Abiotic stress, ethylene, fruit senescence, ionotropic glutamate receptors, NADPH oxidase, RBOH, ROP GTPases, ROS homeostasis.

## Abstract

Apple (*Malus*×*domestica* Borkh) fruits are stored for long periods of time at low temperatures (1 °C) leading to the occurrence of physiological disorders. ‘Superficial scald’ of Granny Smith apples, an economically important ethylene-dependent disorder, was used as a model to study relationships among ethylene action, the regulation of the ROP-GAP rheostat, and maintenance of H_2_O_2_ homeostasis in fruits during prolonged cold exposure. The ROP-GAP rheostat is a key module for adaptation to low oxygen in *Arabidopsis* through Respiratory Burst NADPH Oxidase Homologs (RBOH)-mediated and ROP GTPase-dependent regulation of reactive oxygen species (ROS) homeostasis. Here, it was shown that the transcriptional expression of several components of the apple ROP-GAP machinery, including genes encoding RBOHs, ROPs, and their ancillary proteins ROP-GEFs and ROP-GAPs, is coordinately and negatively regulated by ethylene in conjunction with the progressive impairment of apoplastic H_2_O_2_ homeostatic levels. RNA sequencing analyses showed that several components of the known ROP- and ROS-associated transcriptional networks are regulated along with the ROP-GAP rheostat in response to ethylene perception. These findings may extend the role of the ROP-GAP rheostat beyond hypoxic responses and suggest that it may be a functional regulatory node involved in the integration of ethylene and ROS signalling pathways in abiotic stress.

## Highlight

In cold-stressed apple skins, the ethylene-dependent negative regulation of ROP-GAP rheostat transcripts and apoplastic H_2_O_2_ homeostasis suggests an unprecedented putative crosstalk between ROS and ethylene in abiotic stress.

## Introduction

Cold stress represents a major environmental abiotic challenge for plants and results in severe crop losses, both in the field and after harvesting (Mahajan and Tuteja, 2005). Fruits are artificially subjected to prolonged post-harvest cold storage to extend their marketing period. Even though fruits tolerate exposures to nearly freezing temperatures for relatively long periods of time, after a certain threshold of cold-stress exposure is reached, they undergo a number of cold-induced ‘physiological disorders’, making them unmarketable (Lyons, 1973). Apple scald is a chilling-dependent physiological disorder that is induced in fruits of susceptible apple (*Malus*×*domestica* L. Borkh) cultivars (e.g. Granny Smith) after a minimum period of cold exposure (1–3 months, 1–5 °C) is reached (Watkins *et al.*, 1995). This causes important losses and has initiated research into its underlying mechanisms (reviewed by Lurie and Watkins, 2012). Apple scald results in irregularly shaped necrotic areas on the fruit’s surface, involving hypodermal tissues immediately underneath the epicarp (Bain, 1956). The development of these symptoms is thought to be caused by oxidative reactions, resulting in the production of conjugated trienol oxidative products of the sesquiterpene α-farnesene, which accumulate during storage in response to cold. A burst of H_2_O_2_ production finally leads to lipid peroxidation, cell membrane damage, and cell death (Lurie and Watkins, 2012). Apple scald can be prevented by treatments with the inhibitor of ethylene perception 1-methylcyclopropene (1-MCP), indicating that scald is ethylene dependent (Fan *et al.*, 1999; Rupasinghe *et al.*, 2000; Watkins *et al.*, 2000). However, apple scald can also be fully or partially controlled by the use of the antioxidant diphenylamine (DPA) (Smock 1957; Lau, 1990) indicating that oxidative processes play an important role in its development (Whitaker, 2004). Although several studies have clarified important aspects of scald symptoms development, the molecular factors responsible for its induction are still poorly understood. Many studies have attempted to link the oxidative burst occurring during scald development with the de-regulation of enzymes involved in scavenging of reactive oxygen species (ROS) (Du and Bramlage, 1995; Zubini *et al.*, 2007). However, these and other studies have not revealed a clear relationship between regulation of antioxidant enzyme activity and scald progression. Furthermore, no reports have studied in depth the regulation of ROS homeostasis during the inductive phase of scald, despite the fundamental role played by ROS as signalling molecules in the adaptation to several abiotic stresses (reviewed by Sierla *et al.*, 2013; Baxter *et al.*, 2014; Gilroy *et al.*, 2014). The fine-tuning of ROS levels at diverse subcellular locations evokes and controls local and/or systemic adaptation responses (Baxter *et al.*, 2014), and thus the balance between ROS production and scavenging is likely to play a fundamental role in scald development. The superoxide (O_2_⋅^–^)-producing enzyme NADPH oxidase (termed RBOH in plants, for Respiratory Burst Oxidase Homologue) is a key element in regulating ROS production during adaptation to several environmental stresses including drought, heat, and light intensity (reviewed by Suzuki *et al.*, 2011; Gilroy *et al.*, 2014). RBOH activity and ROS homeostasis are subject to a tightly regulated negative-feedback control, through the so-called ROP-GAP rheostat, which defines a plant’s capacity to adapt to low oxygen availability (Baxter-Burrell *et al.*, 2002). The ROP-GAP rheostat relies on the ROP monomeric small GTPases (Zheng and Yang, 2000; Vernoud *et al.*, 2003). ROPs are molecular switches that, in their GTP-bound active state, positively regulate RBOH activity and superoxide/H_2_O_2_ production. This reaction is negatively regulated by ROP GTPase activating proteins (ROP-GAPs), which inactivate ROPs by enhancing their intrinsic GTPase activity and are transcriptionally induced when H_2_O_2_ levels rise over a certain threshold, thus providing a rheostatic negative-feedback regulatory control (Baxter-Burrell *et al.*, 2002). Based on these data, it was hypothesized that the ROP-GAP rheostat may be a generally conserved regulatory hub for the adaptation of plants to different abiotic stresses. This hypothesis has not been tested further and no data are available to suggest any involvement of ROP proteins in the regulation of cold-stress responses nor any role for ethylene in the regulation of the ROP-GAP rheostat in abiotic stresses in general.

This study identified the components of the *Malus×domestica* (apple) ROP-GAP rheostat, and studied their mode of expression in fruit during prolonged exposure to low temperatures and apple superficial scald induction. The results showed that ethylene negatively regulates the ROP-GAP rheostat of apple fruits and that this negative regulation is associated with the progressive disruption of apoplastic ROS homeostasis during cold exposure. The data suggest that the ethylene-dependent control of the ROP-GAP rheostat may be a previously unidentified element in the loss of cellular ROS homeostasis, thereby potentially leading to enhanced susceptibility to pathophysiological states such as superficial scald.

## Materials and methods

### Sequence identification and analysis

*Arabidopsis thaliana* ROPs, ROP-GEFs, ROP-GAPs, ROP-GDIs, RBOHs, and PLDα were used as BLASTP queries against grape, rice, and poplar sequences in the Ensembl Plants (Kersey *et al.*, 2012) and in the apple genome (Velasco *et al.*, 2010) to retrieve putative orthologues. Sequences were aligned by CLUSTALX (Jeanmougin *et al.*, 1998), the presence for conserved domains was checked, and rooted phylogenetic trees were generated by the neighbour-joining method (Kumar *et al.*, 2008).

### Plant material and treatments

**Fig. 1.**
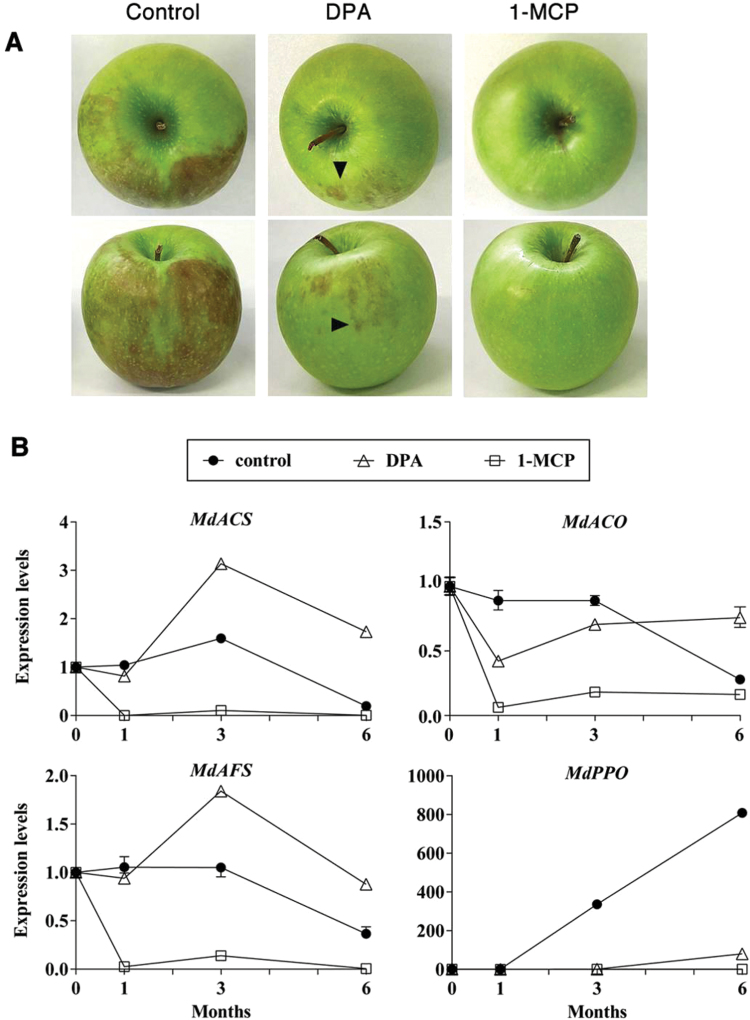
(A) Superficial scald symptoms in control untreated, DPA-treated or 1-MCP-treated Granny Smith apples after 6 months of storage in a controlled atmosphere at 1 °C followed by 8 d at room temperature. Extensive development (more than 50% of the fruit surface) of scald symptoms was evident in 97% of all analysed apples in untreated samples (control). Partial development of symptoms (less than 25% of fruit surface), indicated by arrowheads, was visible (7.4% of all analysed apples) in DPA-treated apples. There was absence of visible scald symptoms in apples pre-treated with 1-MCP. (B) Relative gene expression levels of the two main ethylene biosynthetic marker genes, *MdACS* and *MdACO*, and of the superficial scald marker genes *MdAFS* and *MdPPO*. Filled circles, untreated control apples; open squares, 1-MCP-treated apples; open triangles, DPA-treated apples. Each value represents the average of three independent biological replicates±SD (This figure is available in colour at *JXB* online).

Apple fruits (*Malus*×*domestica*) cv. Granny Smith, were harvested in Trentino Alto-Adige (Italy) in the 2009/2010 and 2010/2011 seasons. Apples were treated or not with 625 ppm m^–3^ of 1-MCP (Rohm and Haas, Mozzate, Italy) or 2000 ppm (w/v) of DPA (Sigma-Aldrich, Milan, Italy) and stored in a controlled atmosphere (0.8% O_2_, 0.8% CO_2_) at 1 °C. Samples were taken at harvest and after 1, 3, and 6 months of storage. After storage, apples were kept at room temperature for 8 d and the development of superficial scald was scored as the percentage of fruits showing symptoms on more than 25% of the fruit’s surface (for DPA, apples displaying less than 25% were counted) (Fig. 1A). Apple peels were excised after exit from storage and frozen in liquid nitrogen. For ethylene treatments, apples were treated with 100 ppm or kept in air for 4 and 24h at 20 °C in sealed glass jars under continuous flushing. For diphenyleneiodonium (DPI) treatment, apples were vacuum infiltrated with 100 µM DPI (Sigma-Aldrich) and 0.001% Tween 20, or with 0.001% Tween 20 as a control.

### RNA extraction, cDNA synthesis, and real-time quantitative reverse transcription PCR (RT-qPCR)

Total RNA was extracted, reverse transcribed, and used for real-time qPCR experiments as described by Nonis *et al.* (2012). Selective primers were constructed on divergent putative 3’-untranslated regions (UTRs) determined by sequence alignments and poly(A)-tail prediction (HCpolyA; Milanesi *et al.*, 1996). Primers (Supplementary Table S1, available at *JXB* online) were designed with Primer3 (Rozen and Skaletsky 2000) and tested with PRaTo (Nonis *et al.*, 2011). Data were elaborated with DataAssist (Applied Biosystems, Monza, Italy) and normalized to Md_8283:1:a (Botton *et al.*, 2011) using the Livak and Schmittgen (2001) method. General good-practice guidelines for RT-qPCR (Udvardi *et al.*, 2008; Remans *et al.*, 2014) were adopted and primers efficiencies (Supplementary Table S1) were calculated and considered for differentially expressed genes as described by Nonis *et al.* (2012). The choice of RT-qPCR for gene expression analyses was based on suggestions reported by Nonis *et al.* (2014). All analyses were carried out on three independent biological replicates for each time point and experimental condition.

### RNA sequencing (RNA-seq) analysis and data processing

RNA samples were processed using TruSeq (Illumina, San Diego, CA, USA) by a third-party service (IGA Technologies Services, Udine, Italy). Raw data were aligned on the *Malus*×*domestica* coding sequence (http://www.phytozome.net/apple.php; release 196) and processed using CLC Bio Genomics Workbench software (CLC Bio, Denmark). Hierarchical clusters and heatmaps were generated using R (R Core Team, 2013) with the package gplots (http://CRAN.R-project.org/package=gplots) from RNA-seq data normalized on data at harvest before transformation into logarithmic values. Data were filtered for genes with at least five counts on at least three samples and normalized with the full quantile method (EDASeq; Risso *et al.*, 2011). Differentially expressed genes were obtained by modelling the count with a negative binomial distribution (edgeR; Robinson *et al.*, 2010). *P* values were adjusted to control the false discovery rate (FDR) (Benjamini and Hochberg, 1995). For gene co-expression, the Pearson correlation coefficient was calculated adopting a cut-off of 0.95 and applied to a dataset of 21 independent RNA-seq experiments (three biological replicates obtained at harvest, at 1 and 6 months of cold storage in control conditions, or after 1-MCP or DPA treatment). Gene Ontology Enrichment Analysis was carried out with BiNGO (Maere *et al.*, 2005) with FDR-adjusted *P* values (Hypergeometric test).

### Determination of MDA, H_2_O_2_, and low-molecular-weight thiol levels

For quantitation of MDA, frozen apple peels (~0.2g) were ground to a powder under liquid nitrogen and homogenized in 25 vols (w/v) of 80:20 (v/v) ethanol:water, followed by centrifugation at 3000*g* for 10min at 4 °C (Hodges *et al.* 1999). The supernatant was centrifuged (20 000*g*, 10min, 4 °C), filtered (0.45 µm Micro-spin^®^) (Grace Davison Discovery Science, Illinois, USA) and 50 µl of the supernatant was mixed with 445 µl of thiobarbituric acid (TBA) reagent (Sigma-Aldrich) and 5 µl of 2mM butylated hydroxytoluene (BHT) (dissolved in methanol) (Sigma-Aldrich) as described by Lärstad *et al.* (2002). The TBA-MDA adduct content was analysed by HPLC with fluorescence detection. TBA for calibration was prepared as described by Fukunaga *et al.* (1998).

The spectrophotometric quantification of H_2_O_2_ was carried out by means of a PeroXOquant Quantitative Peroxide Assay kit (Pierce, Rockford, IL USA), following the manufacturer’s instructions. HPLC analysis of H_2_O_2_ levels was performed by determination of resorufin resulting from the peroxidase-catalysed reaction between H_2_O_2_ and the fluorogenic substrate Amplex^®^ Red (Invitrogen, Molecular Probes, Eugene, USA), dissolved in DMSO to a 10mM final concentration (Zhou *et al.*, 1997). Frozen tissue powder was homogenized in 1ml of 50mM sodium phosphate buffer, pH 7.4, held on ice for 5min, and centrifuged at 10 000*g* (10min, 4 °C). The supernatant was filtered as described above and 50 µl were mixed mixed with 50 µl of Amplex^®^ Red Hydrogen Peroxide/Peroxidase Assay kit working solution, incubated at 30 °C for 30min in the dark and the reaction was terminated by addition of 100 µl of 10mM HCl, 4mM BHT in ethanol. Derivatized MDA and resorufin formation were quantified using a Hewlett-Packard series 1100 HPLC system equipped with Simmetry Shield RP8 column (4.6×250mm, 5 µm; Waters Corp., Milford, MA, USA) and a Simmetry C_8_ column (4.6×250mm, 5 µm; Waters Corp.), respectively, and a multiple wavelength detector (Agilent Technologies, formerly Hewlett-Packard GmbH, Germany). Fluorescence excitation and emission were 560–585nm for MDA and 532–553nm for resorufin. Data were integrated using the Hewlett-Packard ChemStation software (version A.10.02). External standard calibration curves were in the range 0.1–10 nmol ml^–1^ for MDA and 0.1–5 nmol ml^–1^ for resorufin and were linear over the concentration range (*r* ^2^≥0.99). Low-molecular-weight thiol extraction and quantitative evaluation were carried out according to Masi *et al.* (2002).

H_2_O_2_ was localized by visualizing cerium perhydroxide precipitates formation after the reaction between CeCl_3_ and H_2_O_2_ by transmission electron microscopy as described by Bestwick *et al.* (1997).

## Results

### Identification of the apple ROP-GAP rheostat components

In order to identify the genetic components of the apple ROP-GAP rheostat, sequences that encode ROPs in *A. thaliana* (Vernoud *et al.*, 2003; Molendijk *et al.*, 2004), their positive and negative regulatory proteins ROP-GEFs (GDP/GTP exchange factors) (Berken *et al.*, 2005; Gu *et al.*, 2006) and ROP-GAPs (GTPase activating proteins) (Wu *et al.*, 2000), respectively, and ROP-GDIs (GDP dissociation inhibitors, sequestering ROPs in their inactive state) (Berken and Wittinghofer, 2008), together with RBOHs (Torres *et al.*, 1998) and PLDα (Qin and Wang, 2002), the latter responsible for the generation of phosphatidic acids regulating RBOH activity (Zhang *et al.*, 2009), were used as queries to search the *Rosaceae* database (http://www.rosaceae.org). Conserved domains were identified on the predicted protein sequences to select *bona fide* proteins for each family in the apple genome (Figs S1–S6, available at *JXB* online). Distance trees were obtained for members of all families in *Arabidopsis*, poplar (*Populus tricocarpa*), grape (*Vitis vinifera*) and rice (*Oryza sativa*) found in the Ensembl Plants database (http://plants.ensembl.org/index.html) (Figs S7–S12, available at *JXB* online). Apple sequences were renamed according to the most similar genes in *Arabidopsis*. Tissue-specific expression was determined by RT-qPCR to identify transcribed genes (Supplementary Fig. S13 and Supplementary Table S2, available at *JXB* online). In all, 10 ROPs, 14 ROP-GEFs, 10 ROP-GAPs, seven ROP-GDIs, seven RBOHs, and four PLDα *bona fide* encoding genes were identified in the *Malus*×*domestica* genome (described in detail in Supplementary Tables S3–S9, available at *JXB* online).

### Ethylene negatively regulates expression of the apple ROP-GAP rheostat genes in fruit epidermal and hypodermal tissues during cold storage

Apples that had been treated before cold storage with the scald-preventing agents 1-MCP or DPA (Lurie and Watkins, 2012) were analysed and the effects of treatments on the occurrence of scald symptoms were evaluated. After 6 months of storage, 97% of untreated (control) apples underwent scald development. This percentage was reduced to 7.4% in DPA-treated apples and to 0.3% in 1-MCP-treated apples (Fig. 1A, Supplementary Table S10, available at *JXB* online). The expression of the ethylene biosynthetic genes encoding 1-aminocyclopropane-1-carboxylic acid (ACC) synthase (*MdACS 5B*) and oxidase (*MdACO*) (Dal Cin *et al.*, 2005), and of the markers of scald development α-farnesene synthase (*MdAFS*) (Lurie *et al.*, 2005) and polyphenol oxidase (*MdPPO*) (Boss *et al.*, 1995) was used to test the effectiveness of treatments. In peels of untreated control apples, the expression of *MdACS*, *MdACO*, and *MdAFS* remained steadily high until 3 months of cold storage and started to decline thereafter, while *MdPPO* transcripts underwent a progressive increase (up to ~1000-fold) throughout cold storage (Fig. 1B). 1-MCP treatment downregulated *MdACO*, *MdACS*, *MdAFS*, and *MdPPO* transcript abundance to basal levels, consistent with the inhibition of ethylene perception. DPA treatment resulted in an inhibition of *MdPPO* transcript accumulation for the first 3 months of cold storage, which was partially overcome after 6 months, while it exerted a stimulatory effect on the transcription of *MdACS* and *MdAFS* genes from 3 months of storage onwards. The partial and complete suppressive effects exerted by DPA and 1-MCP, respectively, on *MdPPO* transcript accumulation were consistent with the magnitude of superficial scald development and with a role for *MdPPO* expression as a proxy for scald induction.

**Fig. 2.**
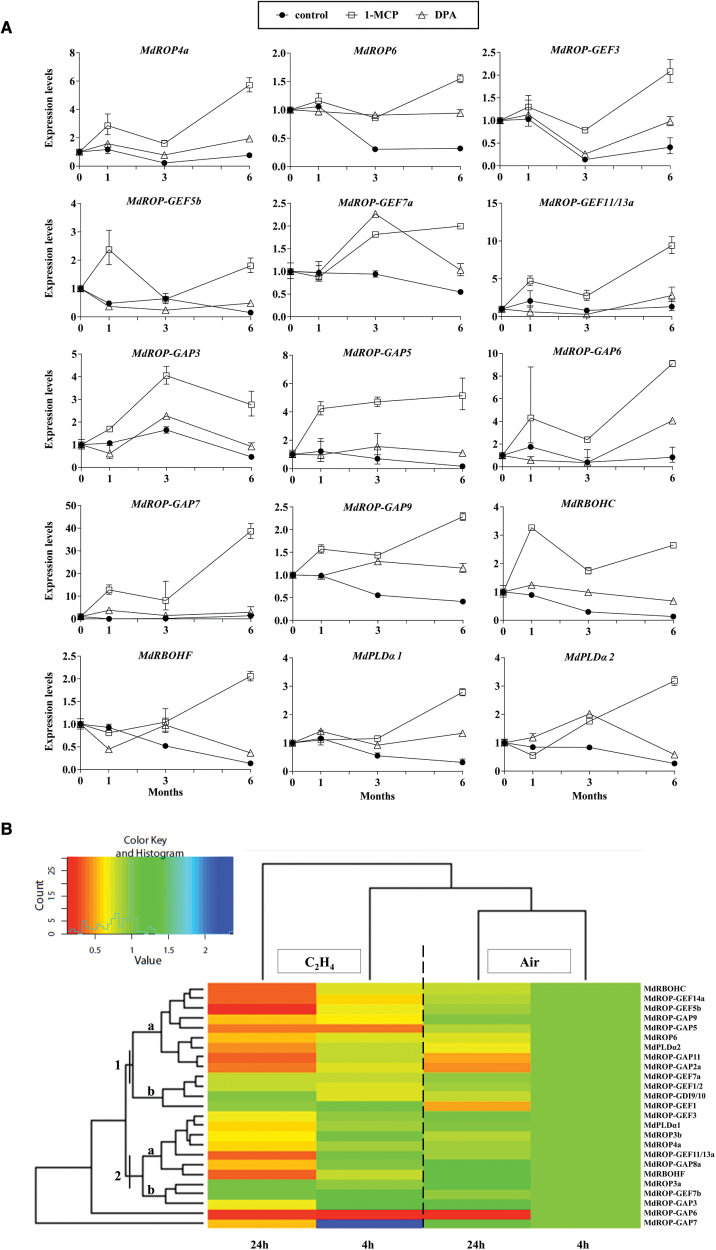
(A) Relative transcriptional expression levels of the ROP-GAP rheostat-encoding genes in cold-stored apples. Gene expression levels were evaluated by real-time RT-qPCR on peels from untreated (control, filled circles), 1-MCP-treated (open squares), or DPA-treated (open triangles) Granny Smith apples at harvest (0) and after 1, 3, and 6 months of cold storage (1 °C) in a controlled atmosphere. Each value represents the average of three independent biological replicates±SD. (B) Heatmap showing the effect of ethylene treatment on the expression of the apple ROP-GAP rheostat-encoding genes. Clustering of the genes was obtained from expression data by real-time RT-qPCR on peels of apples treated with 100 ppm (v/v) of ethylene (C_2_H_4_) or with air for 4 or 24h. Color-coded scale fromn left to right indicates downregulation, no variation, and upregulation relative to control apples maintained in air for 4h (This figure is available in colour at *JXB* online).

Expression analyses on the *Malus*×*domestica* ROP-GAP rheostat genes by RT-qPCR revealed no major differences between treatments for several genes (Supplementary Fig. S14, available at *JXB* online) while, conversely, for a number of genes, a downregulation trend of expression in control untreated apples was evident, starting after 1 month of cold storage. This trend was reversed by 1-MCP treatment, which resulted in the particularly obvious transcriptional de-repression of the *MdROP4a*, *MdROP6*, *MdROP-GEF3*, *MdROP-GEF5b*, *MdROP-GEF7a*, *MdROP-GEF11/13a*, *MdROP-GAP5*, *MdROP-GAP9*, *MdRBOHC*, *MdRBOHF*, and *MdPLDα1* genes (Fig. 2A). Treatment with DPA resulted, for most genes, in a similar expression pattern but with an intermediate de-repressive effect (e.g. evident for *MdROP4a*, *MdROP6*, *MdROP-GAP6*, *MdROP-GAP9*, and *MdRBOHC*) (Fig. 2A). The 1-MCP-dependent de-repression was already evident for some genes after 1 month of cold exposure, pinpointing a subset of early-responsive genes (*MdROP4a*, *MdROP-GEF5b*, *MdROP-GEF11/13a*, *MdROP-GAP3*, *MdROP-GAP5*, *MdROP-GAP7*, *MdROP-GAP9*, and *MdRBOHC*) (Fig. 2A). These transcriptional changes were confirmed by RNA-seq analyses (Supplementary Table S11, available at *JXB* online) and by RT-qPCR on a second independent set of samples (harvest 2010–2011) (Supplementary Fig. S15, available at *JXB* online).

The coordinated negative action of ethylene on the expression of the genes encoding the apple ROP-GAP rheostat machinery was further confirmed by RT-qPCR on peels of apples subjected to short-time treatments with a saturating concentration (100 ppm, v/v) of ethylene for 4 and 24h. Indeed, the expression of a group of early-responsive genes (cluster 1a in Fig. 2B, including *MdROP6*, *MdROP-GEF5b*, *MdROP-GEF14a*, *MdROP-GAP5*, *MdROP-GAP9*, and *MdRBOHC*) was readily downregulated by ethylene after 4h and further downregulated after 24h. Transcription of a second cluster of later-responsive genes downregulated only after 24h of treatment was identified (cluster 2a in Fig. 2B, comprising *MdROP3b*, *MdROP4a*, *MdROP-GEF3*, *MdROP-GEF11/13a*, *MdROP-GAP8a*, *MdRBOHF*, and *MdPLDα1*). Overall, this ethylene-dependent two-step downregulation was in agreement with the time course of de-repression by 1-MCP during cold storage.

### Ethylene induces lipid peroxidation and loss of ROS homeostasis in skins of cold-stored apples

**Fig. 3.**
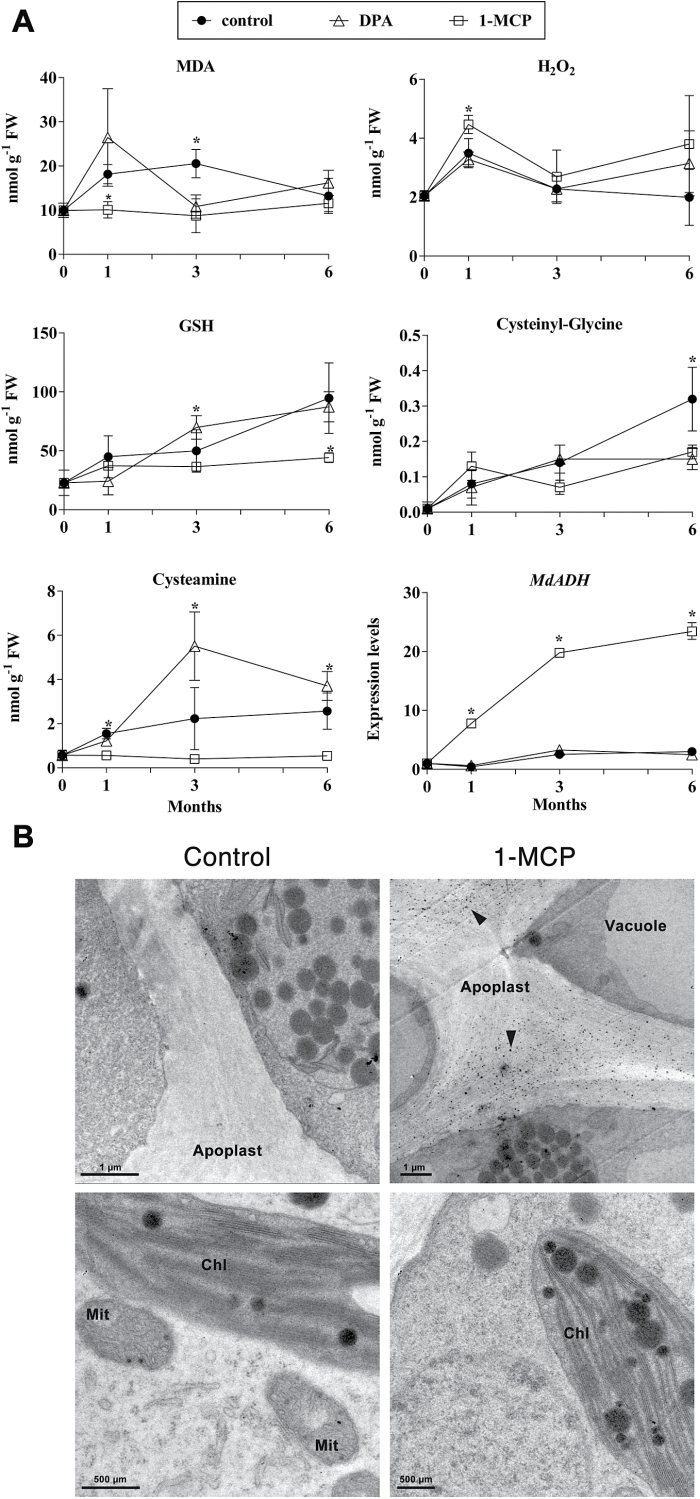
(A) Levels of MDA, H_2_O_2_, and GSH and of its metabolites in peels of cold-stored apples at harvest (0) and after 1, 3, and 6 months of cold storage. Filled circles, control apples; open squares, 1-MCP-treated apples; open triangles, DPA-treated apples. Each value represents an average of four or three (RT-qPCR on *MdADH1*) independent biological replicates±SD. Asterisks indicate significantly different values (*t*-test analysis, *P<*0.05). (B) Cytochemical localization of H_2_O_2_ by cerium perhydroxide precipitation in response to ethylene inhibition by 1-MCP-treated (right panels) and in untreated (left panels) apples, cold stored for 3 months. Arrowheads in the upper right panel show abundant precipitates of apoplastic H_2_O_2_ in peels of 1-MCP-treated apples. Lower panels show the absence of detectable intracellular signals in both conditions. Mit, mitochondria; Chl, chloroplast.

Changes in the transcription rate of apple ROP-GAP rheostat gene members are expected to affect H_2_O_2_ homeostasis and oxidative stress. Therefore, the effect of ethylene perception was tested during cold storage to determine its effect on the levels of H_2_O_2_ and malonyldialdehyde (MDA), a by-product of lipid peroxidation (Frenkel and Neff, 1983), as well as on the levels of glutathione (GSH) and its metabolites. MDA levels were significantly higher in peels of untreated (control) apples between 1 and 3 months of cold storage, indicating the occurrence of oxidative stress in these samples, in comparison with the lower levels maintained throughout the experiment in 1-MCP-treated samples (Fig. 3A and Supplementary Table S12, available at *JXB* online). A similar behaviour was observed in response to DPA treatment, with the exception of a transient non-significant increase after one month of storage. H_2_O_2_ content, measured by two independent techniques (spectrophotometry and HPLC analyses) generally remained higher (significantly after one month of cold storage) and more stable in 1-MCP treated samples, compared to control and, to a lesser extent, to DPA treated samples. H_2_O_2_ content in peels of control untreated apples decreased over time (Fig. 3A, Supplementary Fig. S16, and Supplementary Table S13, available at *JXB* online). The levels of total GSH, one of the main antioxidants for maintenance of H_2_O_2_ homeostasis through the Halliwell–Asada cycle (Noctor and Foyer, 1998; Asada, 1999; Rahantaniaina *et al.*, 2013), were higher in control and DPA-treated apples, after 6 months reaching values significantly higher than the generally lower basal levels maintained in 1-MCP-treated samples (Fig. 3A, Supplementary Table S14, available at *JXB* online). The higher content of GSH in untreated apples was paralleled by higher cysteinyl-glycine levels, the product of GSH metabolism by γ-glutamyl transferase activity, while both 1-MCP and DPA treatments inhibited its formation (Fig. 3A, Supplementary Table S15, available at *JXB* online). The levels of cysteamine, another thiol related to GSH levels that may be involved in oxidative stress and the senescence processes (Moreno *et al.*, 2008), remained at significantly lower and basal levels in response to 1-MCP, and, to a lesser extent, DPA treatment (Fig. 3A, Supplementary Table S16, available at *JXB* online). The transcript abundance of the apple *ADH1 (Alcohol Dehydrogenase*) gene, shown in *Arabidopsis* to be modulated by the ROP-GAP rheostat in response to hypoxia by finely tuned H_2_O_2_ levels (Baxter-Burrell *et al.*, 2002), was greatly upregulated in samples treated with 1-MCP, compared with control and DPA-treated apples (Fig. 3A). The subcellular localization of H_2_O_2_, detected by means of cerium perhydroxide precipitation (Bestwick *et al.*, 1997), revealed remarkably higher levels of apoplastic H_2_O_2_ in peels of 1-MCP-treated fruits in comparison with control fruits, while no differences were observed in the cytoplasm and organelles (Fig. 3B). Overall, both the total content and localization of H_2_O_2_ pointed to an ethylene-dependent progressive loss of apoplastic H_2_O_2_ homeostasis along with cold storage, consistent with the parallel downregulation of several components of the ROP-GAP rheostatic machinery.

**Fig. 4.**
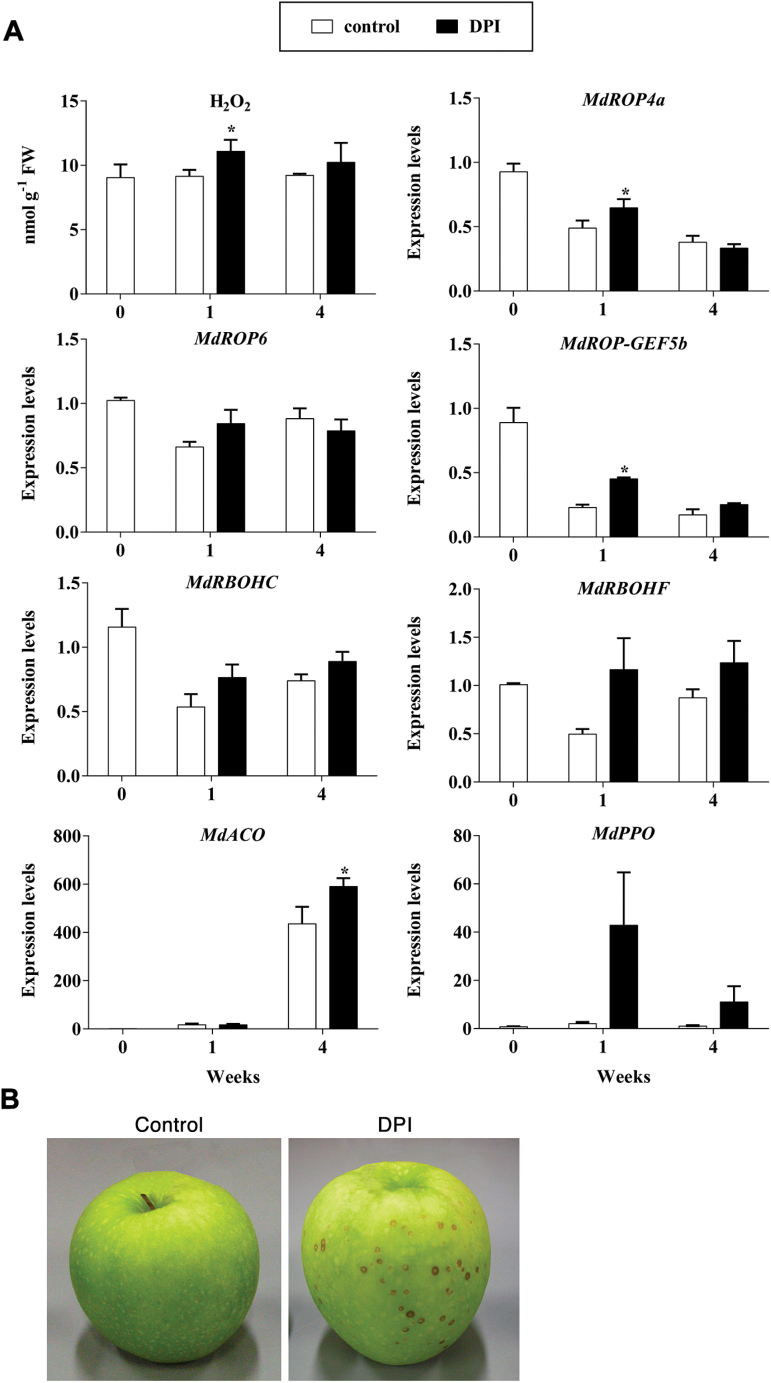
(A) Effects of treatment with the NADPH oxidase inhibitor DPI (100 µM) on H_2_O_2_ levels and on the expression of genes encoding components of the apple ROP-GAP rheostat (*ROP4a*, *ROP6*, *ROP-GEF5b*, *RBOHC*, and *RBOHF*), at harvest (0) and after 1 or 4 weeks of cold storage (1 °C). *MdACO* and *MdPPO* transcript levels (RT-qPCR) were used as markers of the induction of ethylene biosynthesis and oxidative stress, respectively. Each value represents the average of three independent biological replicates±SD. Asterisks indicate significantly different values obtained by *t*-test analysis (*P<*0.05). (B) Development of superficial necrotic lesions on peels (lenticels) of apples treated with DPI occurring after 4 weeks of cold storage (This figure is available in colour at *JXB* online).

To test whether H_2_O_2_ in apple skins is indeed under the regulation of a rheostatic control through the ROP-GAP machinery, apples were subjected after harvest to treatment with 100 μM DPI, an inhibitor of NADPH oxidase activity, followed by cold storage for up to 4 weeks. The expression levels of the ROP-GAP rheostat-encoding genes (chosen from the most responsive from previous data) were investigated along with H_2_O_2_ quantitation. DPI treatment resulted in a significant transient increase of H_2_O_2_ after 1 week of cold storage, which levelled off after 4 weeks reaching values similar to those found in control apples (Fig. 4A). This was paralleled by the increased expression in DPI-treated apples of the *MdROP4a*, *MdROP6*, *MdROP-GEF5b*, *MdRBOH-C* and *MdRBOH-F* genes (Fig. 4A) (but not of *ROP-GAP5*, *ROP-GAP9*, and *ROP-GEF3*; Supplementary Fig. S17, available at *JXB* online). The significant upregulation of *ROP4a* and *ROP-GEF5b* transcripts indicated a compensatory response to transiently lowered NADPH oxidase activity due to DPI inhibition and suggest a negative-feedback control of their expression. Treatment with DPI resulted in transcript accumulation of the scald marker *MdPPO*, peaking after 1 week of cold storage (Fig. 4A), and in the induction of scald-like necrotic lesions on skin lenticels after 4 weeks (Fig. 4B).

### Ethylene-dependent transcriptional rewiring of the apple ‘ROS gene network’ and of ROS- and ROP-GAP rheostat-associated signalling pathways

**Fig. 5.**
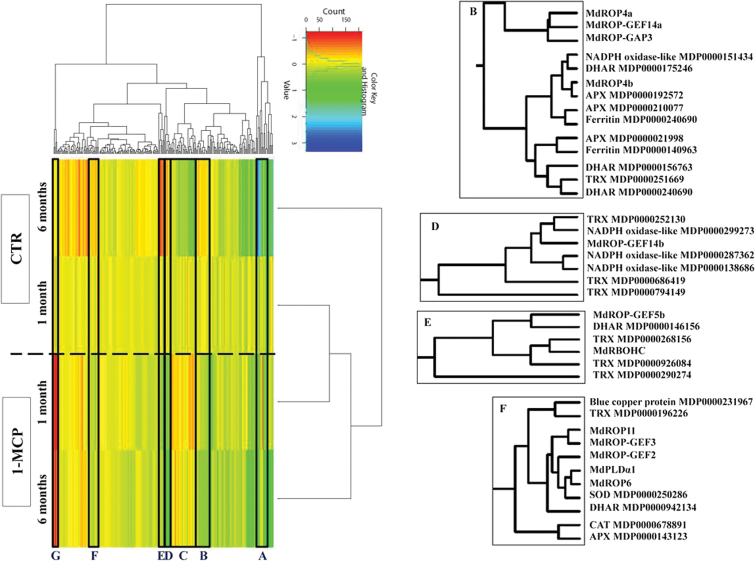
Heatmap showing the expression clustering of genes composing the apple ‘ROS gene network’ and the apple ‘ROP-GAP rheostat’. RNA-seq data obtained from peel samples taken after 1 or 6 months of cold storage, untreated (CTR) or treated with 1-MCP, were used. Colour codes were assigned on the base of normalized log-scaled RPKM (Reads per kb per million mapped reads) values: Red: down-regulation; Green to blue: up-regulation; Yellow: no variation. Clusters of co-expressed genes are boxed. Clusters A, C, and G: genes downregulated in samples treated with 1-MCP; clusters B, D, E, and F: genes de-repressed by 1-MCP treatment. Only clusters B, D, E, and F are expanded for simplicity to permit reading of names of genes co-expressed with the members of the ROP-GAP rheostat (This figure is available in colour at *JXB* online).

Evidence from *Arabidopsis* suggests that different ROS at defined subcellular locations induce specific transcriptional signatures composing the ‘ROS gene networks’ (Mittler *et al.*, 2004). The significant ethylene-dependent changes in apoplastic H_2_O_2_ levels may have consequences for the regulation of the apple ‘ROS gene networks’. By using the identified sequences from *A. thaliana* (Mittler *et al.*, 2004) to query the *Rosaceae* database, the apple ‘ROS gene network’ was characterized and shown to include 316 genes (Supplementary Table S17, available at *JXB* online). By analysing RNA-seq data on samples taken at harvest and after 1 or 6 months of storage, either treated with 1-MCP or not, co-regulated transcriptional signatures could be identified between the genes composing the apple ‘ROS gene network’ and the ‘ROP machinery’. The transcription of several genes involved in the ascorbate–glutathione cycle together with the genes encoding the ROP-GAP rheostat was altered in response to the block of ethylene perception. Three clusters (A, C, and G; Fig. 5) included those genes for which an upregulation of transcript abundance during cold storage was repressed by 1-MCP. These clusters did not include the genes comprising the apple ROP-GAP rheostat, excepted for *MdRBOHD* (data not shown). Instead, the four clusters B, D, E, and F included the apple ROP-GAP rheostat components and the ‘ROS network’ genes whose expression was coordinately downregulated by ethylene in peels of control untreated apples along with those in cold storage and de-repressed by the inhibition of ethylene action those obtained by 1-MCP (Fig. 5). These data, confirming independently those obtained by RT-qPCR, showed the coordinated transcriptional de-repression of *MdROP4a*, *MdROP-GEF14a*, and *MdROP-GAP3* with three dehydroascorbate reductase (DHAR)- and three ascorbate peroxidase (APX)-encoding genes together with three genes encoding thioredoxins (TRXs), involved in the protection of thiol groups from H_2_O_2_ action (Buchanan and Balmer, 2005). Similarly, *MdROP6*, *MdROP-GEF2*, *MdROP-GEF3*, and *MdPLDα1* grouped in cluster F together with genes encoding proteins involved in the detoxification of H_2_O_2_: a Cu/Zn SOD, a CAT, a TRX, an APX, and a DHAR. Finally, three genes encoding ferric-chelate reductases and NADPH oxidase-like proteins were found to be co-regulated with three *TRX* genes and *MdROP-GEF14b* in cluster D, while three *TRX* genes and one *DHAR* gene were co-regulated with *MdRBOHC* and *MdROP-GEF5b* in cluster E (Fig. 5). These changes represent ethylene-dependent transcriptional signatures revealing H_2_O_2_ sensing and/or regulated metabolism through the specific coordinated regulation of genes encoding enzymes of the ascorbate–glutathione cycle (Rahantaniaina *et al.*, 2013), along with those encoding the ROP-GAP rheostat.

The crosstalk between the ROP-GAP rheostat, ROS homesostasis, and ethylene signalling is further supported by untargeted analyses of RNA-seq data highlighting the prominent transcriptional differences that are induced early or repressed by ethylene in cold-stressed apple skins. After 1 month of cold storage, approximately 200 genes were differentially expressed (with a 5-fold induction/repression threshold) between 1-MCP-treated and untreated samples and were assigned to the ‘regulatory’ category by Mapman (Thimm *et al.*, 2004). Among these, several factors could be linked to ROP and ROS signalling (Table 1A). 1-MCP treatment resulted in the significant de-repression of genes encoding a Feronia-like and an RBK2-like kinase, core components of the ROP signalling network in *Arabidopsis* (Molendijk *et al.*, 2008; Duan *et al.*, 2010), and of a gene encoding a C_2_C_2_(Zn) DOF zinc finger transcription factor (*MEE47*) related to ROP10-mediated signalling in *Arabidopsis* (Xin *et al.*, 2005) (Table 1A and Supplementary Table S18, available at *JXB* online). The transcription of several transcription factors of the AP2–EREBP family was upregulated, linked to the regulation of redox homeostasis, such as the Redox Responsive Transcription Factor 1 (RTTF1, Khandelwal *et al.*, 2008), or to abscisic acid (ABA)-mediated adaptation to cold stress, such as some members the DREB subfamily (TINY2-like DREB subfamily A-4 and CBF4, DREB1D subfamily A-1) (Knight *et al.*, 2004). A gene encoding a Heat Shock Factor A2-like (HSFA2) protein was also found to be upregulated, described by Miller and Mittler (2006) as one of the most highly responsive genes to H_2_O_2_ and co-regulated with RTTF1 (Mehterov *et al.*, 2012). Conversely, the transcription of another member of the AP2 group, a putative ABA repressor (ABR1), was significantly downregulated together with two genes encoding WRKY-like transcription factors (*WRKY40* and *WRKY70*), both ABA negative regulators in *Arabidopsis* (Shang *et al.*, 2010; Li *et al.*, 2013) (Table 1A and Supplementary Table S18). RT-qPCR analyses on a selection of these genes confirmed the RNA-seq data (Supplementary Fig. S18, available at *JXB* online). To investigate further the crosstalk between the ROP-GAP rheostat-dependent H_2_O_2_ signalling and ethylene action, the co-expressed transcriptional network of the *MdRBOHC* gene was identified, the most abundantly expressed NADPH oxidase-encoding gene in apple skins. *MdRBOHC* transcript abundance displayed a clear early downregulation in response to ethylene, an effect that was fully reversed by 1-MCP treatment and partially reversed by DPA (Fig. 2A, Supplementary Table S11), making it the most relevant candidate putatively responsible for the ethylene-dependent progressive loss of apoplastic H_2_O_2_ homeostasis in cold-stressed apples. By exploring a dataset of 21 RNA-seq experiments, 109 genes were identified that were highly co-regulated with *MdRBOHC* (Pearson correlation coefficient of expression >0.95). Gene Ontology (GO) enrichment analysis enabled the identification of several factors involved in glutamate perception and glutamate gated-channel activity (Table 1B and Supplementary Table S19, available at *JXB* online). In fact, five genes encoding putative clade 3 ionotropic glutamate receptors (iGLuR3.2, -3.3, and -3.6), acting in *Arabidopsis* as amino acid-gated Ca^2+^ channels (Michard *et al.*, 2011; Forde, 2014), together with a gene encoding an ACT repeat-containing protein probably involved in glutamine signalling (Sung *et al.*, 2011), were de-repressed by 1-MCP along with *MdRBOHC* (Table 1B).

**Table 1.** (A) Regulatory genes differentially expressed in response to inhibition of ethylene perception by 1-MCP in apple peels after 1 month of cold storage. (B) MdRBOHC co-expressed gene list including five genes encoding ionotropic glutamate receptors and highlighting enrichment for the GO term ‘Excitatory extracellular ligand-gated ion channel activity’ For (A), the statistical significance (*P* values) of differential expression for pairwise comparisons is given in Supplementary Table S18. Columns from left to right report: description of the encoded protein, the *Rosaceae* database ID of the encoding gene, the closest *Arabidopsis* homologue, fold change of transcript abundance found between 1-MCP and untreated control samples, putative functional/regulatory process played by the closest *Arabidopsis* homologue, and corresponding reference. TF, transcription factor; PK, protein kinase. (A) Genes differentially expressed in response to inhibition of ethylene perception by 1-MCP.

Description	Rosaceae ID	Closest *Arabidopsis* Homologue	Fold change	Function/regulatory process	References
**TFs**					
AP2/EREBP	*mdp0000175375*	*AT4G34410 – RRTF1 (Redox Responsive TF 1); ERF4*	25.19	Regulation of ROS homeostasis	Khandelwal *et al.* (2008)
AP2/EREBP	*mdp0000242979*	*AT5G64750 – ABR1 (ABA Repressor1*)	–84.53	Induced by ABA, cold, drought, and wounding	Pandey *et al.* (2005)
AP2/EREBP	*mdp0000297646*	*AT5G25190 – ESE3 (Ethylene and Salt Inducible 3*)	–58.27	Response to ethylene, drought, and salt	Zang *et al.* (2011)
AP2/EREBP	*mdp0000652413mdp0000790788*	*AT5G11590 – TINY2, DREB subfamily A-4*	10.06 7.43	Induced by ABA, cold, drought, and wounding	Wei *et al.* (2005)
AP2/EREBP	*mdp0000198054*	*AT5G51990 – CBF4, DREB1D subfamily A-1*	27.1	Response to cold and ABA	Knight *et al.* (2004)
C2C2(Zn) DOF zinc finger	*mdp0000170286*	*AT4G00950 – MEE47 (maternal effect embryo arrest 47*)	10.09	ROP10-dependent ABA Signalling	Xin *et al.* (2005)
HSFA2, Heat Shock TFA2	*mdp0000194672*	*AT2G26150 – ATHSFA2 (Heat Shock Factor A2*)	6.69	Co-regulated with RRTF1 induces tolerance to ROS	Mehterov *et al.* (2012)
WRKY 40	*mdp0000177906*	*AT1G80840 – WRKY40*	–37.51	Negative regulator of ABA response	Shang *et al.* (2010)
WRKY 70	*mdp0000175240*	*AT3G56400 – WRKY70*	–38.63	Negative regulator of ABA response	Li *et al.* (2013)
**PK/phosphatase**					
Receptor-like kinase VII	*mdp0000493959*	*AT3G51550 – FER* (*FERONIA*)	14.58	ROP-GEF regulator, repressing ABA responses	Duan *et al.* (2010); Yu *et al.* (2012)
Receptor-like cytosolic kinase VI	*mdp0000287486*	*AT3G05140 – RBK2 (Rop Binding protein Kinase 2*)	17.96	ROP-binding protein kinase	Molendjik *et al*. (2008)

**Table:** (B) MdRBOHC co-expression transcriptional network: enriched GO terms.

Description	Rosaceae ID	Closest *Arabidopsis* Homologue	Fold change	Function / regulatory process	References
**Excitatory extracellular ligand-gated ion channel activity: ionotropic glutamate receptors (GLRs**)					
GLR3.6	*mdp0000487438 mdp0000432508 mdp0000462878*	*AT3G51480 – ATGLR3.6*	4.52 3.33 3.90	Amino-acid-gated Ca^2+^ transport	Michard *et al.* (2011); Forde (2014)
GLR3.2/3.3	*mdp0000265636mdp0000313051*	*AT4G35290 – ATGLR3.2AT1G42540 – ATGLR3.3*	3.59 2.84	Amino-acid-gated Ca^2+^ transport	Michard *et al.* (2011); Forde (2014)
ACT domain protein	*mdp0000257180*	*AT2G39570 – ACT domain- containing protein*	2.23	Glutamine signalling	Sung *et al.* (2011)

## Discussion

### Ethylene downregulates the ROP-GAP rheostat and impairs apoplastic ROS homeostasis in apple peel during cold storage

This study adopted apple scald induction as a model system to investigate the regulation of the ROP-GAP rheostat and ROS signalling in relation to prolonged cold stress and ethylene action in fruits. In fact, the regulation of genes of the ROP machinery during fruit ripening and senescence is poorly characterized in general and only a few reports are available (Falchi *et al.*, 2010). By using known components from *A. thaliana*, the constituents of the apple (*Malus*×*domestica* Borkh) ROP-GAP rheostat machinery were identified together with those of rice (*O. sativa*), poplar (*P. thricocarpa*), and grape (*V. vinifera*). Analyses of transcript abundance (by both RT-qPCR and RNA-seq) during prolonged cold stress, following either ethylene inhibition or treatments with exogenous ethylene, showed that ethylene exerts a negative effect on the transcript levels of several components of the apple ROP-GAP rheostat. Ethylene downregulated the expression of genes encoding ROP-GEFs (*MdROP-GEF3*, *5a*, *11/13a*) and ROP-GAPs (*MdROP-GAP3*, -*5*, and -*9*), proteins required for the activation and deactivation of ROPs, respectively (Fig. 2, Supplementary Table S11). Also, the expression of two ROP-encoding (*MdROP4a* and *MdROP6*) and two RBOH-encoding (*MdRBOHC* and *MdRBOHF*) genes was significantly downregulated, suggesting that ethylene action may indeed result in an overall disruption of apoplastic H_2_O_2_ homeostasis in cold-exposed apple peels. This was confirmed by the fact that skins of 1-MCP-treated apples maintained higher steady-state levels of H_2_O_2_, which otherwise displayed a progressive decline along with cold storage in the absence of treatments (Fig. 3A and Supplementary Fig. S16, Supplementary Table S13). This difference appeared to be due to a significantly higher apoplastic H_2_O_2_ level (Fig. 3B). It is remarkable to note that lipid peroxidation in 1-MCP-treated (and to a lesser extent in DPA-treated) apples, evaluated by the MDA content as well as the levels of cysteamine, a thiol known to be associated with senescence processes (Moreno *et al.*, 2008), remained at basal levels throughout the entire experimental period. This evidence supports the hypothesis that the higher homeostatic apoplastic levels of H_2_O_2_ maintained when ethylene signalling was blocked were not detrimental for cells and may act instead as finely controlled signals perceived as second messengers for stress adaptation. The transcriptional activation of the *ADH1* gene in 1-MCP-treated apples in the absence of a hypoxic stimulus (Fig. 3A), taken as a ROP-GAP and H_2_O_2_-dependent marker as shown in *Arabidopsis* (Baxter-Burrell *et al.*, 2002), supports such a conclusion. Further support comes from the simultaneous de-repression of several apple ROP-GAP encoding genes, which may be upregulated as a negative-feedback system required to control ROP activity and H_2_O_2_ levels. The H_2_O_2_-dependent transcriptional activation of *Arabidopsis ROP-GAP4* is required to lock ROPs into a negative-feedback cycle to ensure maintenance of homeostasis, by avoiding the build-up of an oxidative burst through an otherwise uncontrolled rise of ROS levels, necessary for the proper regulation of *ADH* gene expression and acclimation to low oxygen (Baxter-Burrell *et al.*, 2002). The activation of such a homeostatic loop in apple skins in response to 1-MCP is in agreement with the hypothesis that apoplastic H_2_O_2_ indeed may act as a signalling molecule for stress adaptation and that its levels are under continuous control. This latter aspect is also confirmed by the upregulation of *MdROP4a* and *MdROP-GEF5b* transcripts induced by the use of the NADPH oxidase inhibitor DPI (Fig. 4A). This suggests that, as soon as a perturbation of NADPH oxidase activity takes place, the genes responsible for its reactivation are upregulated, conceivably as a compensatory response, in agreement with the concept of a finely tuned rheostatic system.

The ethylene-dependent control of H_2_O_2_ homeostasis in apple skins seems to involve the regulation of GSH metabolism, the main cellular antioxidant. Indeed, the lower total content of GSH found in 1-MCP-treated apples in the presence of higher apoplastic H_2_O_2_ levels is in agreement with the Halliwell–Asada cycle (Noctor and Foyer, 1998; Asada, 1999) and indicates a faster turnover of GSH to keep H_2_O_2_ within a homeostatic concentration range to prevent a ROS burst. Consistently, the lower content of the GSH metabolite cysteinyl-glycine and of cysteamine, which can be related to GSH levels (McCoy, 2012), in DPA- and 1-MCP-treated apple skins, suggests that the metabolic degradation of GSH is inhibited in response to both treatments (Fig. 3A). Therefore, when ethylene perception is blocked, the higher level of apoplastic H_2_O_2_ in apple peels seems to be homeostatically controlled through increased scavenging at the expense of GSH.

### Ethylene and ROP signalling crosstalk in skins of cold-stored apples: evidence from rewiring of ROS and ROP-GAP rheostat-associated transcriptional networks

In the presence of the significantly higher apoplastic H_2_O_2_ levels found in skins of 1-MCP-treated apples, rather divergent ROS transcriptional signatures should be expected. Studies in *Arabidopsis* have demonstrated that diverse ‘ROS transcriptional signatures’ reveal the activation of adaptive responses to different ROS molecules in various subcellular compartments (Mittler *et al.*, 2004). This study tested this hypothesis by identifying the apple ‘ROS transcriptional network’ according to that characterized in *Arabidopsis* by Mittler *et al.* (2004) and by mining RNA-seq data. The block of ethylene perception resulted in rewiring of the apple ‘ROS transcriptional network’, highlighting the co-regulation of the ROP-GAP rheostat gene expression with that of genes involved in H_2_O_2_ metabolism, such as APXs and DHARs, or in the protection of thiol groups from H_2_O_2_ attack, such as TRXs (Rahantaniaina *et al.*, 2013) (Fig. 5). It is noteworthy that, while the expression of different sets of APX-encoding genes was upregulated in both control and 1-MCP-treated apples, that of DHAR-encoding genes was found to be specifically upregulated in response to the inhibition of ethylene action. This is a signature of the activation of H_2_O_2_ metabolism through oxidation of ascorbate to dehydroascorbate (DHA) and the subsequent regeneration of ascorbic acid through the reduction of DHA by DHARs at the expense of GSH (Rahantaniaina *et al.*, 2013). This shift is reported as the prominent pathway regulating intracellular GSH metabolism for H_2_O_2_ homeostatic control and H_2_O_2_-mediated signalling (reviewed in detail by Rahantaniaina *et al.*, 2013), in agreement with the lower level of GSH found in DPA- and 1-MCP-treated apples with respect to control samples.

RNA-seq data also indicated that the finely tuned ethylene-regulated and ROP-GAP-dependent apoplastic H_2_O_2_ homeostasis could be perceived and translated into signalling cascades leading to cold-stress sensitivity or adaptation. ROS perception and regulation of the redox state was evidenced by the 1-MCP-induced de-repression of *RTTF1* (Khandelwal *et al.*, 2008) and *HSFA2*, the latter reported to be the *Arabidopsis* heat-shock factor most highly responsive to H_2_O_2_ (Miller and Mittler, 2006) and co-regulated with *RTTF1* (Mehterov *et al.*, 2012). Several differentially regulated genes could be linked to ROP or ROS signalling and/or to ROP/ROS-mediated ABA responses (Table 1A). In fact, 1-MCP treatment resulted in the de-repression of two genes encoding a Feronia-like and an RBK2-like kinase, respectively. Feronia is a member of the *Catharantus roseus* family of receptor-like kinases (CrRLKs) (Cheung and Wu, 2011) and a negative regulator of ABA responses through the direct interaction with and phosphorylation of ROP-GEFs in *Arabidopsis* (Yu *et al.*, 2012). Feronia is also an important factor in auxin-mediated root hair development through the regulation of RBOH activity (Duan *et al.*, 2010) and a negative regulator of ethylene responses in hypocotyls (Deslauriers and Larsen, 2010). The cytosolic receptor-like kinase *RBK2*, identified as ROP-binding kinase 2 (Molendijk *et al.*, 2008), is also part of the ROP signalling pathway, although its precise role in ethylene signalling is currently unknown. The ethylene-dependent transcriptional regulation of the apple genes encoding Feronia- and RBK2-like kinases further strengthens the convergence of ethylene, ROS, and ROP signalling pathways and suggests that these kinases may be key points for crosstalk between hormonal and ROP signalling networks. This is also supported by the transcriptional de-repression induced by 1-MCP of a gene encoding a C_2_C_2_(Zn) DOF zinc finger transcription factor related to ROP10-mediated ABA signalling in *Arabidopsis* (Xin *et al.*, 2005). In addition, crosstalk with ABA signalling was also evidenced by the ethylene-dependent transcriptional repression of members of the DREB subfamily of transcription factors involved in ABA-mediated adaptation to cold stress (Knight *et al.*, 2004), and by the transcriptional activation of a gene encoding ABR1, a repressor of ABA responses, together with two genes encoding WRKY-like transcription factors (WRKY40 and WRKY70) (Table 1A), both negative regulators of ABA responses in *Arabidopsis* (Shang *et al.*, 2010; Rushton *et al.*, 2012). Overall, these data may support ROP-dependent ethylene–ABA crosstalk, considering that different ROP monomeric GTPases have been reported to be negative regulators of the ABA response (Zheng *et al.*, 2002; Li *et al.*, 2012).

**Fig. 6.**
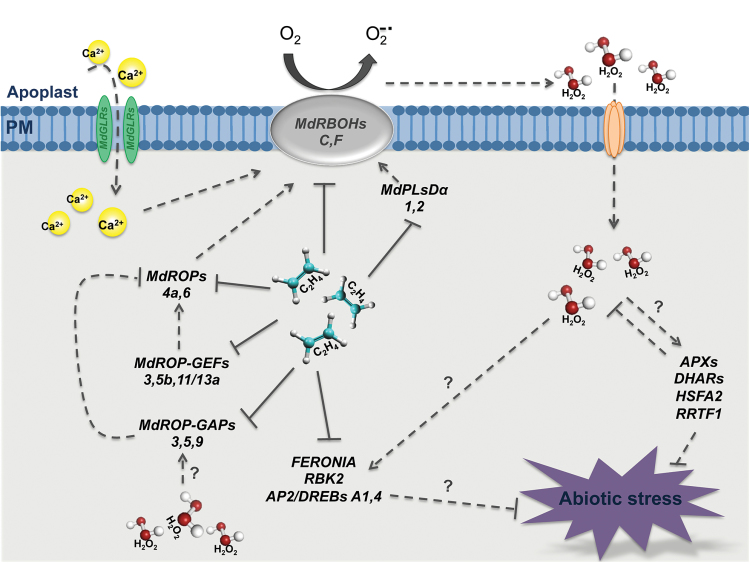
Schematic hypothetical model of the ethylene-dependent regulation of the apple ROP-GAP rheostat in dermal and hypodermal tissues of apple fruits subjected to cold storage. Solid lines indicate the negative regulation exerted by ethylene on the transcript levels of different components of the apple ROP-GAP rheostat (ROPs, ROP-GAPs, ROP-GEFs, and RBOHs) and of glutamate receptor-encoding genes, linked with a progressive loss of H_2_O_2_ apoplastic homeostasis. Dotted arrows indicate the transcriptional de-repression induced by the inhibitor of ethylene action 1-MCP. Question marks indicate the genes that may hypothetically be regulated as a consequence of H_2_O_2_ homeostatic level maintenance, such as genes encoding proteins involved in H_2_O_2_ metabolism (APXs and DHARs), redox responsive transcriptions factors (HSFA2 and RTTF1), regulatory kinases (Feronia and the Rop-binding kinase RBK1) and genes involved in the negative-feedback regulation of ROP4a and ROP6 GTPases (ROP-GAP3, - 5, and -9) required for maintenance of H_2_O_2_ homeostasis. (This figure is available in colour at *JXB* online).

The characterization of the transcriptional neighbourhood of *MdRBOHC*, the prevalent ethylene-responsive NADPH oxidase-encoding gene expressed in apple skins, allowed us to further explore the connections between ethylene and ROP-GAP rheostat signalling, opening up interesting new perspectives. The *MdRBOHC* gene was remarkably found to be co-expressed with five genes encoding putative clade 3 ionotropic glutamate receptors (iGluR3.2, -3.3, and -3.6). In *Arabidopsis*, clade 3 glutamate receptors act as amino acid-gated cation channels mediating calcium fluxes (Michard *et al.*, 2011; Forde, 2014). Glutamate receptors have been shown to be involved in the regulation of pollen tube growth (Michard *et al.*, 2011), root architecture (Forde 2014), ABA biosynthesis and response, and immune responses (Kang *et al*., 2004, 2006). Their remarkable ethylene-dependent *MdRBOHC* co-regulated expression adds glutamate receptors to the ROP-GAP rheostat signalling pathways and links them to crosstalk with ethylene in the abiotic stress response, an aspect unexplored so far. The upregulation induced by 1-MCP of the five apple glutamate receptors may result in elevated cytoplasmic Ca^2+^ signatures and in the subsequent activation of MdRBOHC activity through binding to EF-hand motifs. This could contribute to a positive-feedback loop in concert with other components of the ROP-GAP rheostat for maintenance of H_2_O_2_ levels (Fig. 6). On the other hand, H_2_O_2_ levels seem to be kept within a homeostatic range, and thus prevented from building up an oxidative burst, by the concerted upregulation of several *MdROP-GAP*s (*GAP3*, -*5*, and -*9*), which may be required for the inactivation of MdROP4a and MdROP6 GTPases through a negative-feedback loop (Fig. 6). These data may suggest a scenario in which RBOH action may generate ROS-dependent signalling signatures in a finely concerted crosstalk with the action of ethylene.

### Conclusion

This study showed that an extended apple ROP-GAP rheostat may be a highly regulated and important signalling hub required for adaptation to cold stress, through the control of apoplastic H_2_O_2_ levels. Ethylene is a central hormonal regulator of this homeostatic system, negatively regulating the maintenance of apoplastic H_2_O_2_ homeostasis in apples subjected to prolonged cold stress and leading to its progressive decay, which may finally terminate with the development of superficial apple scald. This ethylene-dependent regulatory mechanism involves the concerted co-regulation of three interdependent mechanisms: (i) modulation of expression of several members of the ROP-GAP rheostat; (ii) the concomitant rearrangement of H_2_O_2_ metabolism and scavenging through the ascorbate–GSH cycle; and (iii) the gene regulation of glutamate receptor calcium channels. Further biochemical work will be needed to fully determine the role of the ROP-GAP rheostat during fruit storage and, in particular, to clarify how the balance between ROP-GEFs and ROP-GAPs proteins may finally modulate the activation/inactivation ratio of ROPs and RBOHs and how this may be linked to the control of apoplastic H_2_O_2_ homeostasis in response to ethylene perception.

## Supplementary data

Supplementary data are available at *JXB* online.

Supplementary Fig. S1. Alignment of the conserved domains of the apple MdROP proteins.

Supplementary Fig. S2. Alignment of the three PRONE (plant-specific Rop nucleotide exchanger) conserved domains identified within the deduced protein sequences of apple MdROP-GEFs.

Supplementary Fig. S3. Alignment of conserved domains identified within the apple MdROP-GAPs deduced protein sequences.

Supplementary Fig. S4. Conserved GDI-like domains of the apple MdROP-GDI proteins.

Supplementary Fig. S5. Conserved domains of the apple MdRBOH proteins.

Supplementary Fig. S6. Conserved domains of the apple PLDα proteins.

Supplementary Fig. S7. Phylogenetic tree of the ROP proteins from different plant species including apple.

Supplementary Fig. S8. Phylogenetic tree of ROP-GEF proteins from different plant species including apple.

Supplementary Fig. S9. Phylogenetic tree of ROP-GAP proteins from different plant species including apple.

Supplementary Fig. S10. Phylogenetic tree of ROP-GDI proteins from different plant species including apple.

Supplementary Fig. S11. Phylogenetic tree of RBOH proteins from different plant species including apple.

Supplementary Fig. S12. Phylogenetic tree of PLDα proteins from different plant species including the apple candidates.

Supplementary Fig. S13. Tissue-specific expression of the apple ROP-GAP rheostat components.

Supplementary Fig. S14. Regulation of transcriptional expression of the apple ROP-GAP rheostat-encoding genes in peels of apple fruits during cold storage (harvest 2009–2010).

Supplementary Fig. S15. Transcriptional expression of the apple ROP-GAP rheostat-encoding genes in peels of apple fruits during cold storage (harvest 2010–2011).

Supplementary Fig. S16. Spectrophotometric determination of H_2_O_2_ levels in peels of Granny smith apples during cold storage and in response to ethylene inhibition.

Supplementary Fig. S17. Effects of treatments with 100 µM diphenylene iodonium chloride (DPI) on the expression of the genes encoding ROP-GAP5, ROP-GAP9, and ROP-GEF5b in peels of Granny Smith apples.

Supplementary Fig. S18. Effect of cold storage and of 1-MCP or DPA treatments on relative transcript levels of genes involved in ABA and ROP signalling.

Supplementary Table S1. Primers pairs used in this work for RT-qPCR experiments.

Supplementary Table S2. Tissue-specific expression of the genes encoding the apple ROP-GAP machinery.

Supplementary Table S3. Putative ROP-encoding sequences identified in the apple genome.

Supplementary Table S4. Putative ROP-GEF-encoding sequences identified in the apple genome.

Supplementary Table S5. Putative ROP-GAP-encoding sequences identified in the apple genome.

Supplementary Table S6. Putative ROP-GDI-encoding sequences identified in the apple genome.

Supplementary Table S7. Putative RBOH-encoding sequences identified in the apple genome.

Supplementary Table S8. Putative PLDα-encoding sequences identified in the apple genome.

Supplementary Table S9. Overview of genes encoding the ROP-GAP rheostat in different plant species including apple.

Supplementary Table S10. Percentage of healthy and superficially scalded cv. Granny Smith fruits.

Supplementary Table S11. Excel file RNA-seq expression data for the ROP-GAP components in control, 1-MCP- or DPA-treated apple peels during storage.

Supplementary Table S12. Malonydialdehyde (MDA) content in peels of cold stored apples.

Supplementary Table S13. HPLC analysis of H_2_O_2_ content in peels of cold stored apples.

Supplementary Table S14. GSH content in peels of cold stored apples.

Supplementary Table S15. Cysteinyl-glycine content in peels of cold stored apples.

Supplementary Table S16. Cysteamine content in peels of cold stored apples.

Supplementary Table S17. Overview of apple ‘ROS gene network’.

Supplementary Table S18. Excel file showing genes differentially expressed between 1-MCP-treated and control apple skins after 1 month of cold storage.

Supplementary Table S19. Excel file showing GO enrichment analysis of *MdRBOHC* co-expressed genes.

Supplementary Data
