# Supplementary material for: Ethylene negatively regulates transcript abundance of ROP-GAP rheostat-encoding genes and affects apoplastic reactive oxygen species homeostasis in epicarps of cold stored apple fruits
Source: J Exp Bot. 2015 Oct 1;66(22):7255–70. doi: 10.1093/jxb/erv422 (PMC4765793; doi:10.1093/jxb/erv422)
Supplement: Supplementary Data [file supp_66_22_7255__index.html]

Ethylene negatively regulates transcript abundance of ROP-GAP rheostat-encoding genes and affects apoplastic reactive oxygen species homeostasis in epicarps of cold stored apple fruits — Ethylene negatively regulates transcript abundance of ROP-GAP rheostat-encoding genes and affects apoplastic reactive oxygen species homeostasis in epicarps of cold stored apple fruits — Supplementary Data 

# Ethylene negatively regulates transcript abundance of ROP-GAP rheostat-encoding genes and affects apoplastic reactive oxygen species homeostasis in epicarps of cold stored apple fruits

## Supplementary Data

Data files

- Supplementary Data - Supplementary Data
- Supplementary Data - Supplementary Data
- Supplementary Data - Supplementary Data
- Supplementary Data - Supplementary Data
- Supplementary Data - Supplementary Data
