# Supplementary material for: Ethylene negatively regulates transcript abundance of ROP-GAP rheostat-encoding genes and affects apoplastic reactive oxygen species homeostasis in epicarps of cold stored apple fruits
Source: J Exp Bot. 2015 Oct 1;66(22):7255–70. doi: 10.1093/jxb/erv422 (PMC4765793; doi:10.1093/jxb/erv422)
Supplement: Supplementary Data [file supp_erv422_jexbot154500_file001.pdf]

Supplementary Figures

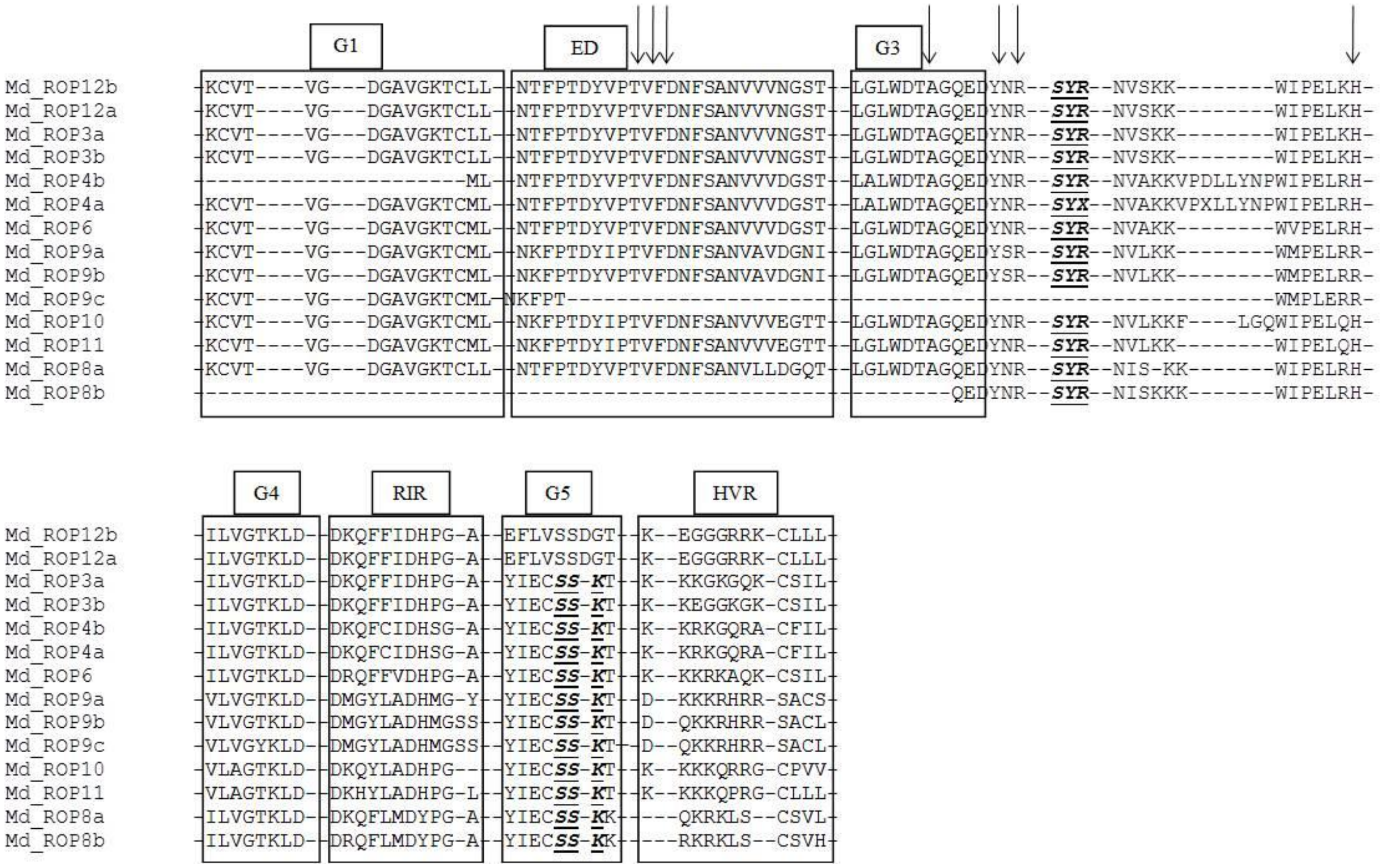

Figure S1. Alignment of the conserved domains of the apple MdROP proteins. The typical conserved domains of ROP proteins (according to Zheng and Yang, 2000 and to Jiang and Ramachandran, 2006) identified within the apple MdROP deduced protein sequences are shown: GTPase domains (G1 and G3 boxes), GDP/GTP-binding domains (G4 and G5 boxes), effector domain (ED), Rho insert region (RIR), putative serine/threonine-dependent phosphorylation sites (motifs SYR and SSK, evidenced with bold underlined character) and the hypervariable region (HVR). Arrows show residues putatively involved in ROP/ROP-GDI interaction.

	P1	P2	P3
MdROP-GEF13a	MKERFSKLLLGEDM-ALALSNAITNL-NIPALRKLDAM-	-KWWKPXVKVPPEGLSDE-	-LDISKIQFNMDVGYAILESYSRVI-
MdROP-GEF13b	MKERFSKLLLGEDM-ALALSNAITNL-NIPALRKLDAM-	-KWWKPMVKVPPEGLSDE-	-LDISKIQFNMDVGYAILESYSRVI
MdROP-GEF11	MKERFSKLLLGEDM-ALALSNAITNL-NVPALRKLDAM-	-KWWKPNVKVPPEGLSDE-	-LDISKIQFNMDVGYAILESYSRVI-
MdROP-GEF12	MKERFAKLLLGEDM-ALALSNAITNL-NIPALRKLDAM-	-KWWLPTPKVPPNGLSDA-	-LDISKIQYNEDVGQAILESYSRIL-
MdROP-GEF7a	MKERFSKLLLGEDM-ALAISNAITNL-NLPALRKLDNM-	-KWWLPVPRVPPGGLHEN-	-LDMSKIQHNKDVGKSILESYSRVL-
MdROP-GEF7b	MKERFSKLLLGEDM-ALAISNAITNL-NLPALRKLDNM-	-KWWLPVPRVPSGGLHKN-	-LDMSKIQYNKDVGKSILESYSRVL-
MdROP-GEF5a	MRERFSKLLLGEDM-AMTISNAITNL-NLPGLRKLDNM-	-KWWLPVPRVAADGLSEN-	-LDTSKIQCNKDVGKSILESYSRVL-
MdROP-GEF5b	MRERFSKLLLGEDM-AMAISNAITNL-NLPALRKLDNM-	-KWWLPVPRVAAEGLSES-	-LDTSKIQCNKDVGKSILESYSRVL-
MdROP-GEF4a	MKERFAKLLLGEDM-AVTISNSITNL-NLPALQKLDAM-	-KWWLPIPCVPPGGLSEK-	-LDTCKIQCNRDVGQAVLESYSRVL-
MdROP-GEF4b	MKERFAKLLLGEDM-AVTISNSITNL-NLPALQKLDAM-	-KWWLPVPCVPPGGLSEK-	-LDTCKIQCNKDVGQSVLESYSRVL-
MdROP-GEF2	MKERFAKLLLGEDM-ALAISNAITNL-NLPAIKKLDAM-	-KWWLPYPKVPPNGLSFE-	-LDMNKIQYNKDVGQSILESYSRVM-
MdROP-GEF1	MKERFAKLLLGEDM-ALAISNAITNL-NLPAIKKLDAM-	-KWWLPYPKVPPNGLSCE-	-LDMNKIQYNKDVGQSILESYSRVM-
MdROP-GEF3	MKERFAKLLLGEDM-ALAISNAITNL-NLPALKKLDAM-	-KWWLPFPKVPSNGLSEN-	-LDMNKIQYNRDVGQSILESYSRVM-
MdROP-GEF14a	MKEKFAKLLLGEDV-ALALSNAITNL-NLPALKKLDSM-	-RWWLPTPQVPATGLSDT-	-LDVTKIQYGKDIGHSILEAYSRVL-
MdROP-GEF14b	---------------------------NLPALKKLDSM-	-RWWLPTPQVPATGLSDT-	-LDVTKIQYGKDIGHSILEAYSRVL-
MdROP-GEF14c	MKEKFAKLLLGEDV-ALALSNAITNL-NLPALKKLDSM-	-RWWLPTPQVPATGLSDT-	-LDVTKIQYGKDIGHSILEAYSRVL-

Figure S2. Alignment of the three PRONE (plant-specific Rop nucleotide exchanger)(P1-P3) conserved domains (Berken *et al*., 2005; Shin *et al.*, 2009; Riely *et al*., 2011) identified within the deduced protein sequences of apple MdROP-GEFs.

	CRIB-like motif	Src homology domain 3-binding motif PXXXXXPXXP	GAP-like domain
Md_ROP-GAP6	-MSRMS--------LF	DR--**P**TELE**P**EV**P**R-	-PTILLMMQERLYSGGGLK-AEGIFRIN-DVHCLAG-
Md_ROP-GAP5	-ISSPSEVRHVSHVTF	DR--**P**TELE**P**EV**P**R-	-PTILLMMQERLYSGGGLK-AEGIFRIN-DVHCLAG-
Md_ROP-GAP3	-IGWPSNVRHITHVTF	DR--**P**VEFEVEI**P**G-	-PTILLLMQERLYSQGGLK-AEGIFRIN-DVHCLSG-
Md_ROP-GAP7	-IGWPTNVQHVTHVTF	DR--**P**VEFEVEV**P**G-	-PTILLLMQERLYSQEGLK-AEGIFRIN-DIHCLAG-
Md_ROP-GAP9	-IGWPSNVRHVAHVTF	DR--**P**VELE**P**EV**P**R-	-PTILILMQRHLYAQGGLQ-AEGIFRIN-DVHCLAG-
Md_ROP-GAP8b	-IGWPSNVRHVAHVTF	DR--**P**VELE**P**EV**P**R-	-PTILILMQRHLYAQGGLQ-AEGIFRIN-DVHCLAG-
Md_ROP-GAP8a	-IGWPSNVRHVAHVTF	DR--**P**VELE**P**EV**P**R-	-PTILILMQRHLYAQGGLQ-AEGIFRIN-DVHCLAG-
Md_ROP-GAP10	-IGWPSNVRHVAHVTF	DR--**P**VELE**P**EV**P**R-	-PTILILMQRHLYAQGGLQ-AEGIFRIN-DVHCLAG-
Md_ROP-GAP2a	-IGWPTNVRHVAHVTF	DR--**P**VEFE**P**EV**P**R-	-PTILLLMQGRLYAEGGLQ-AEGIFRIN-DVHCLAG-
Md_ROP-GAP2b	-IGLPTNVRHVAHVTF	DR--**P**VEFE**P**EV**P**S-	-PTILLLMQGRLYAEGGLQ--EKEFKSF-YSIMISK-
Md_ROP-GAP11	----------------	---------------	----------------------IFASF-FYIFFS–

Md_ROP-GAP6	-LIKAWFRELPTR-LPPTEASLLDWAINLMADVAQNEQHNKMNARNIAMVFAP-
Md_ROP-GAP5	-LIKAWFRELPTR-LPPTEASLLDWAINLMADVAQNEQHNKMNARNIAMVFAP-
Md_ROP-GAP3	-LIKAWFRELPGV-LKPTEAALLDWAVDLMADVVEEEFNKMNARNIAMVFSP-
Md_ROP-GAP7	-LIKAWFRELPGV-LKPTETALLNWAVNLMADVVEEEELNKMNARNIAMVFAP-
Md_ROP-GAP9	-LIKAWFRELPTA-LPPTEAALLDWAVNLMADVAQMEHFNKMNARNIAMVFAP-
Md_ROP-GAP8b	-LIKAWFRELPTA-LPPTEAALLDWAVNLMADVAQMEHHNKMNARNIAMVFAP-
Md_ROP-GAP8a	-LIKAWFRELPTA-LPPTEAALLDWAVNLMADVAQMEHHNKMNARNIAMVFAP-
Md_ROP-GAP10	-LIKAWFRELPTG-LPPTEAALLNWAVNLMADVVEEEEFNKMNPRNIAMVFSP-
Md_ROP-GAP2a	-LIKMSP-------NP----------INTHIAVEE-------------------
Md_ROP-GAP2b	-KSSAWFRELPAG-LPPTEASLLDWAINLMADVVQQEHLNKMNARNIAMVFAP-
Md_ROP-GAP11	-FTKAWFRELPAG-LPLTEVSLLDWAINLMADVVQQEHLNKMNARNIAMVFAP-

Figure S3. Alignment of conserved domains identified within the apple MdROP-GAPs deduced protein sequences: Cdc42/Rac-interacting binding (CRIB) motif, consensus sequence for SCR homology domain 3-binding motif PXXXXPXXP and GAP-like domain (Wu *et al.*, 2000).

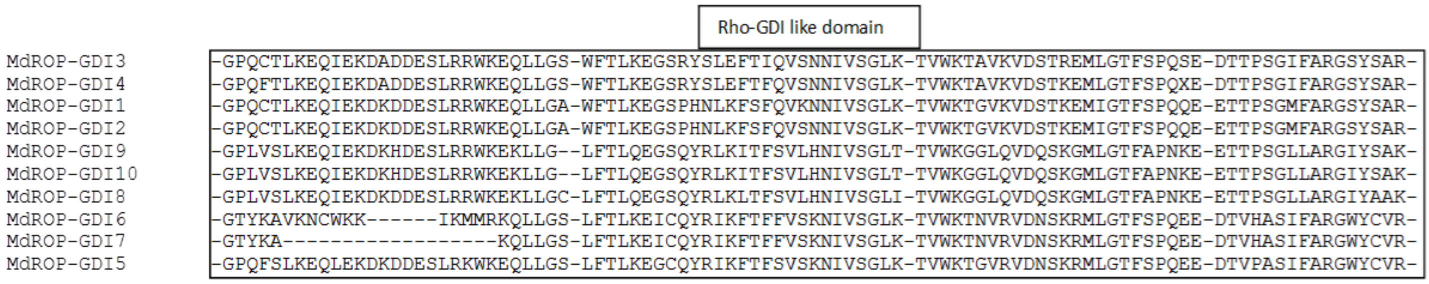

Figure S4. Conserved GDI-like domains of the apple MdROP-GDI proteins. Alignment of the conserved GDI-like domain of deduced protein sequences from apple ROP-GDIs (Berken and Wittinghofer, 2007).

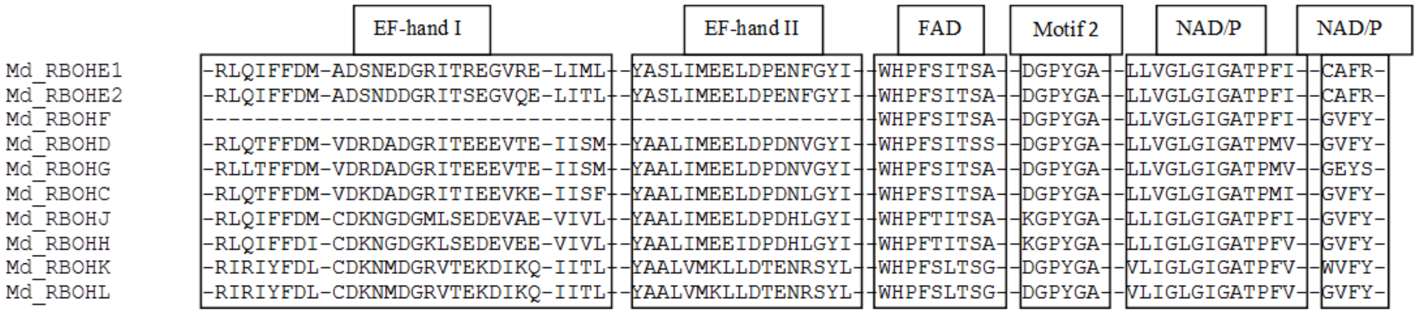

Figure S5. Conserved domains of the apple MdRBOH proteins. Alignment of conserved domains of the apple RBOHs deduced protein sequences showing the typical EF-hand motifs (EF-hand I and II) and nucleotide binding motifs (FAD-isoalloxazine binding site: FAD; Motif 2; NADPH-ribose and NADPH-binding sites: NAD/P; (Keller *et al.*, 1998; Torres *et al.*, 1998; Amicucci *et al.*, 1999).

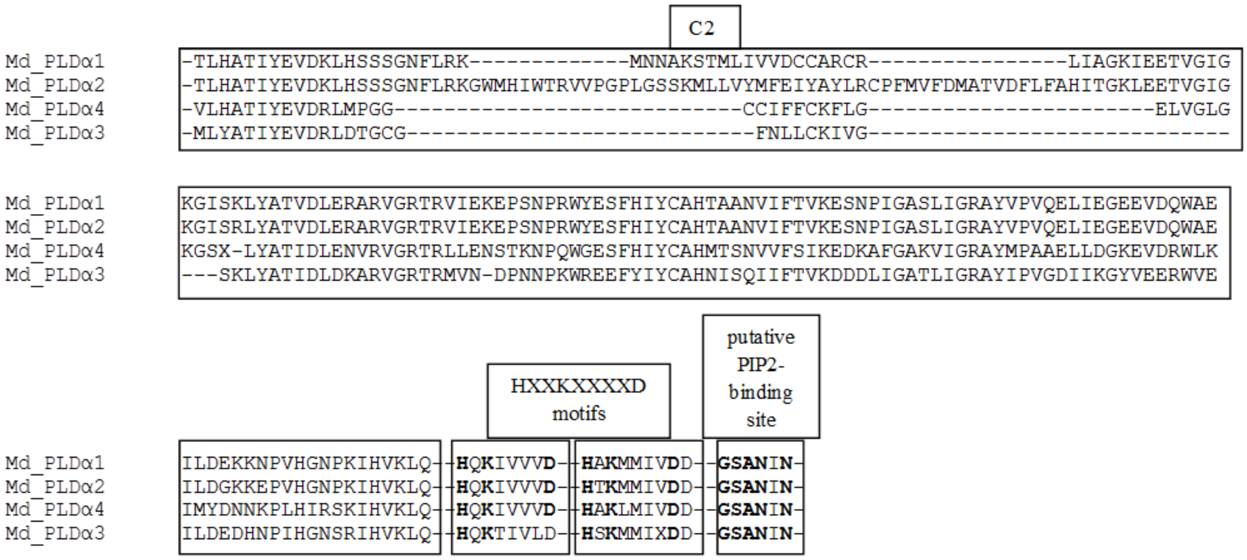

Figure S6. Conserved domains of the apple PLDα proteins. Alignment of conserved domains of the apple PLsDα deduced protein sequences: C2 domain (C2), two HKD motifs (HXXKXXXXXD) and putative PIP2-bnding site (Qin and Wang 2002; Du *et al.*, 2013).

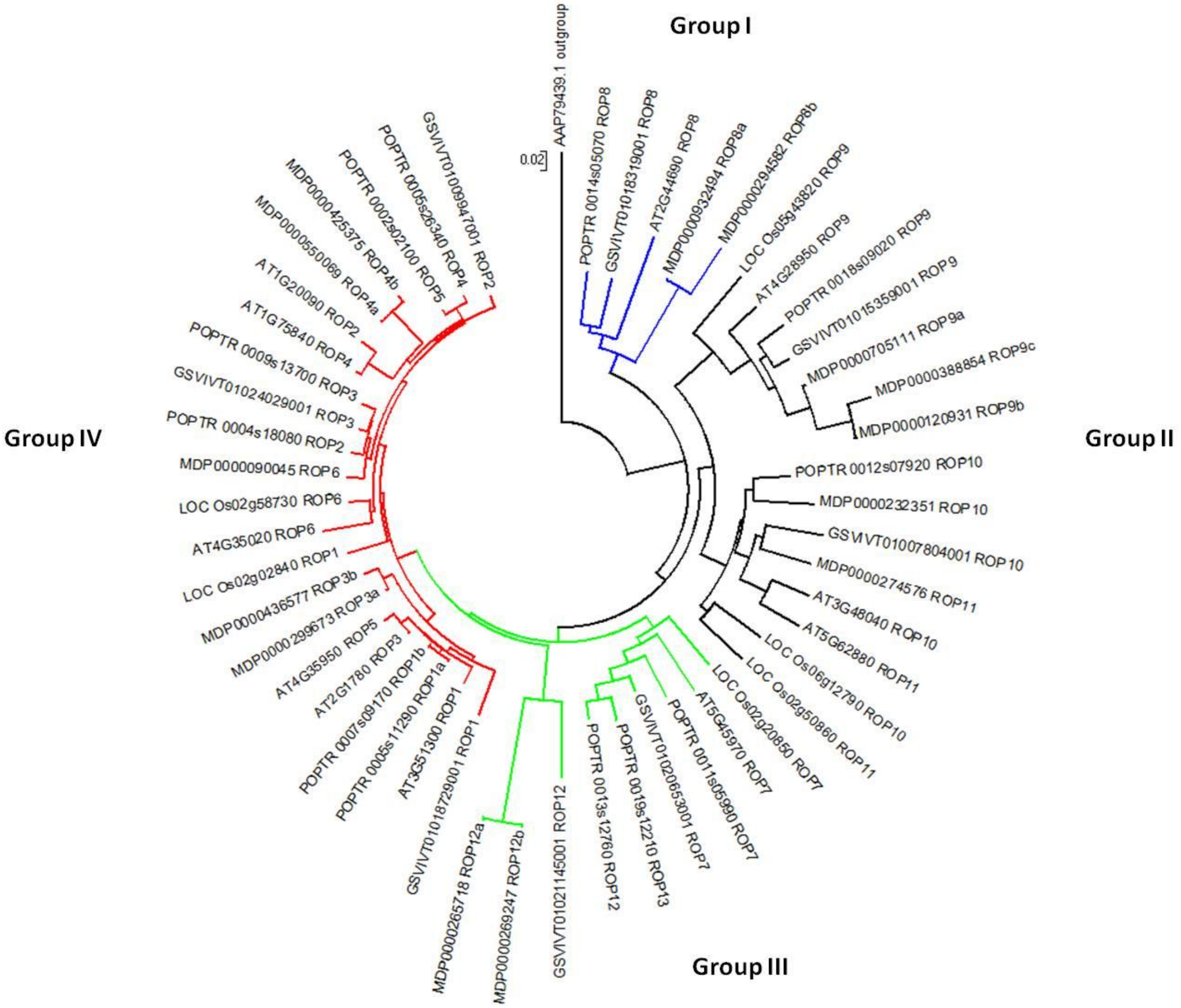

Figure S7. Phenetic tree of the ROP proteins from different plant species including apple. The phenetic tree shows the relationships between the fourteen identified *Malus domestica* MdROP deduced protein sequences (identified in the apple genome, http://www.rosaceae.org, Velasco *et al.*, 2010, and marked by the corresponding MDP number) and those from *A. thaliana* (AGI number), *O. sativa* (LOC Os), *P. thricocarpa* (POPTR) and *V. vinifera* (GSVIV) retrieved from the Ensembl Plants database (http://plants.ensembl.org/index.html). Very short apple ROP sequences were excluded from the analysis. The phenetic tree was constructed by the neighbor-joining method with bootstrapping analysis on the basis of a CLUSTALX alignment (Jeanmougin *et al.*, 1998) and was rooted on the *Trichomonas vaginalis* Rac1-putative protein as outgroup (AAP79439.1). The four groups of ROP sequences identified by Zheng and Yang (2000) are highlighted with different colors.

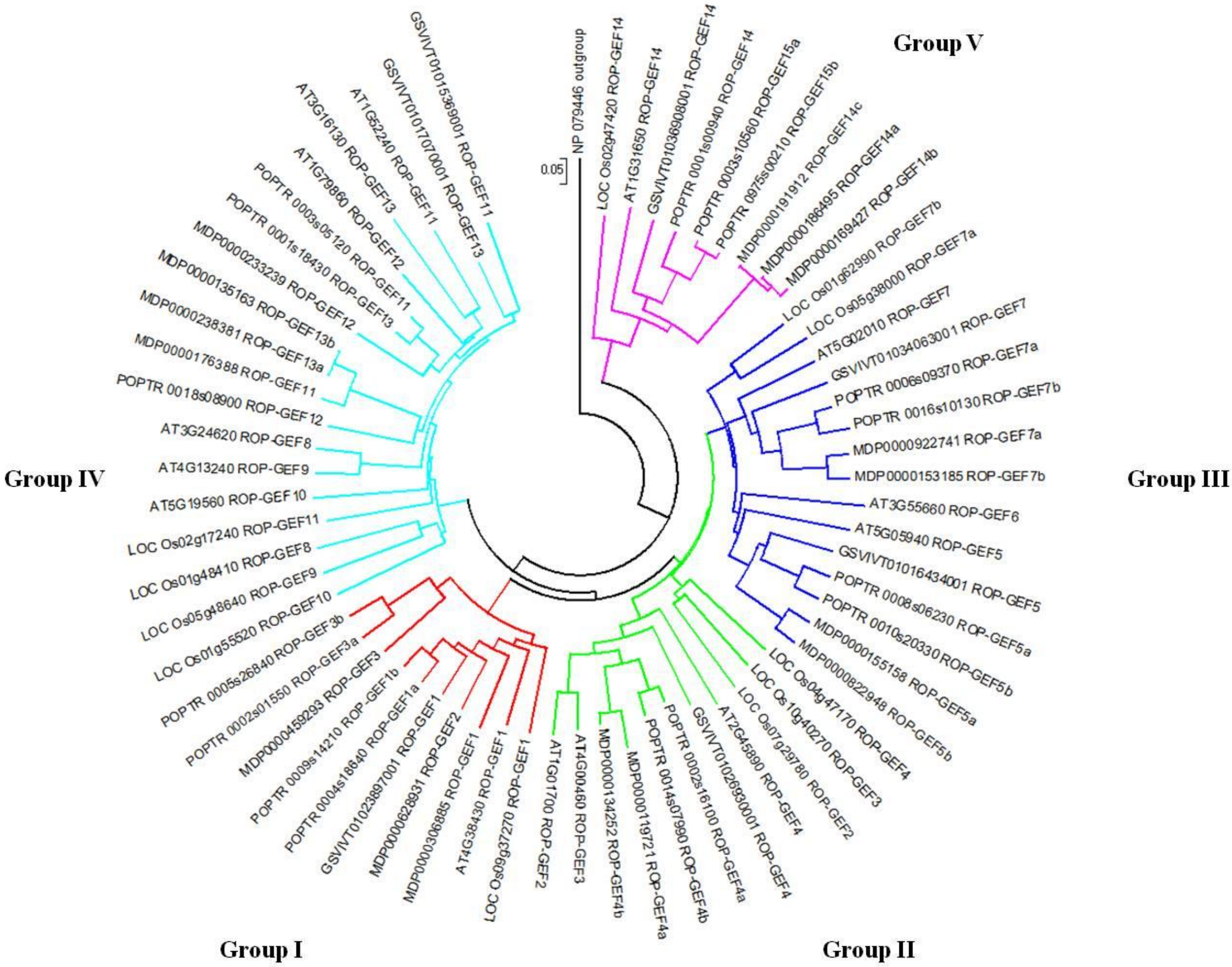

Figure S8. Phenetic tree of ROP-GEF proteins from different plant species including apple. The phenetic tree shows the relationships among the sixteen ROP-GEF sequences identified in the *Malus domestica* genome (http://www.rosaceae.org, Velasco *et al.*, 2010) and those identified in Arabidopsis (*A. thaliana*, identified by AGI number), rice (*O. sativa*, LOC Os), poplar (*P. thricocarpa*, POPTR) and grape (*V. vinifera*, GSVIV) retrieved from the Ensmbl Plants database (http://plants.ensembl.org/index.html). Very short apple ROP-GEF sequences were excluded from the analysis. The phenetic tree was constructed by the neighbor-joining method with bootstrapping analysis on the basis of a CLUSTALX alignment (Jeanmougin *et al.*, 1998) and was rooted on the *Homo sapiens* P-REX2 protein as outgroup (NP_079446). The five groups of ROP-GEF sequences identified by Riely *et al*. (2011) are highlighted with different colors.

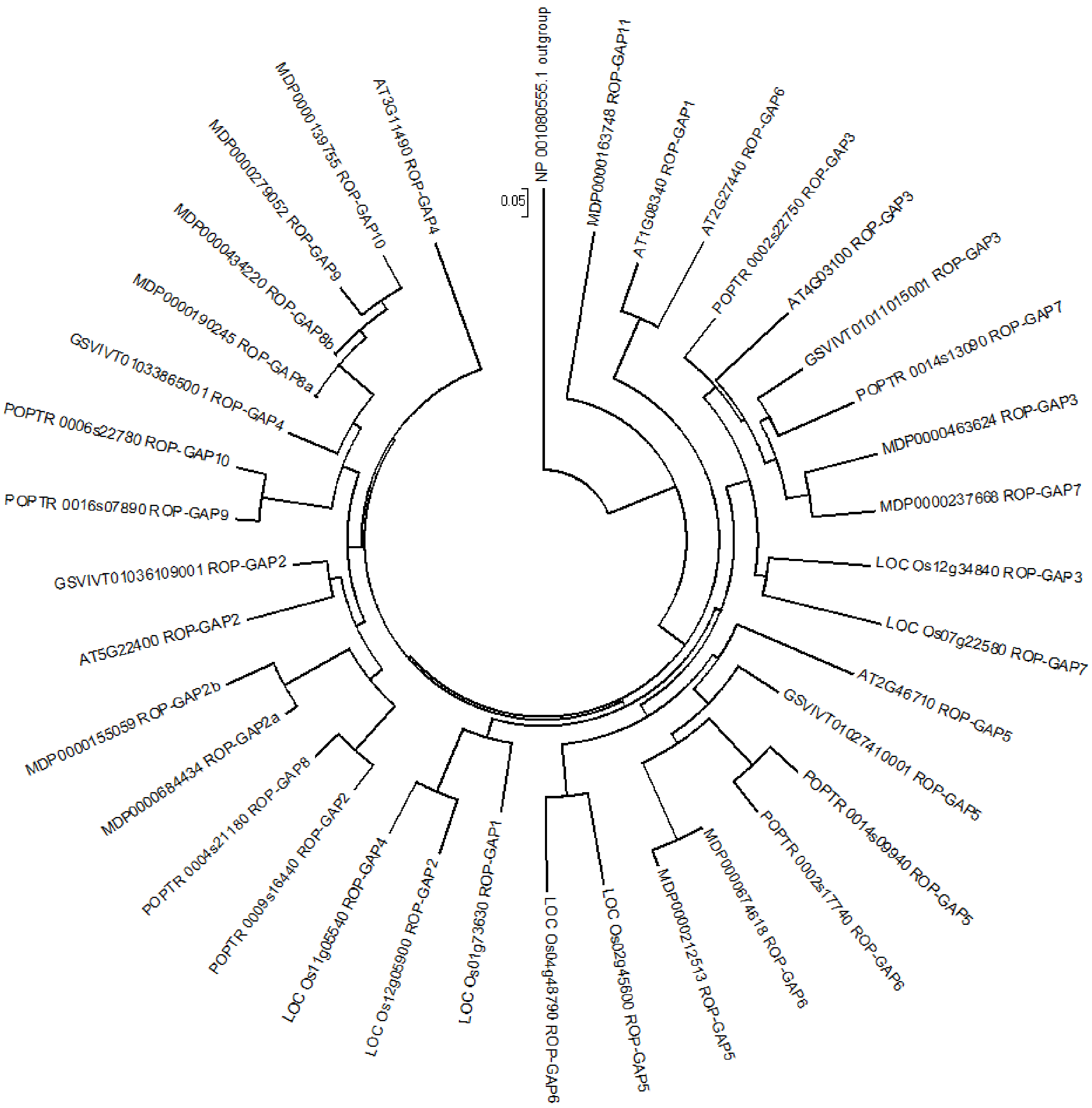

Figure S9. Phenetic tree of ROP-GAP proteins from different plant species including apple. The phenetic tree shows the relationships among the eleven ROP-GAP sequences found to be encoded in the *Malus domestica* genome (http://www.rosaceae.org, Velasco *et al.*, 2010) and those from Arabidopsis (*A. thaliana*, identified by AGI number), rice (*O. sativa*, LOC Os), poplar (*P. thricocarpa*, POPTR) and grape (*V. vinifera*, GSVIV) retrieved from the Ensmbl Plants database (http://plants.ensembl.org/index.html). The phenetic tree was constructed by the neighbor-joining method with bootstrapping analysis on the basis of a CLUSTALX alignment (Jeanmougin *et al.*, 1998) and was rooted on the *Xenopus Laevis* Rho-GAP1 protein as outgroup (NP_001080555).

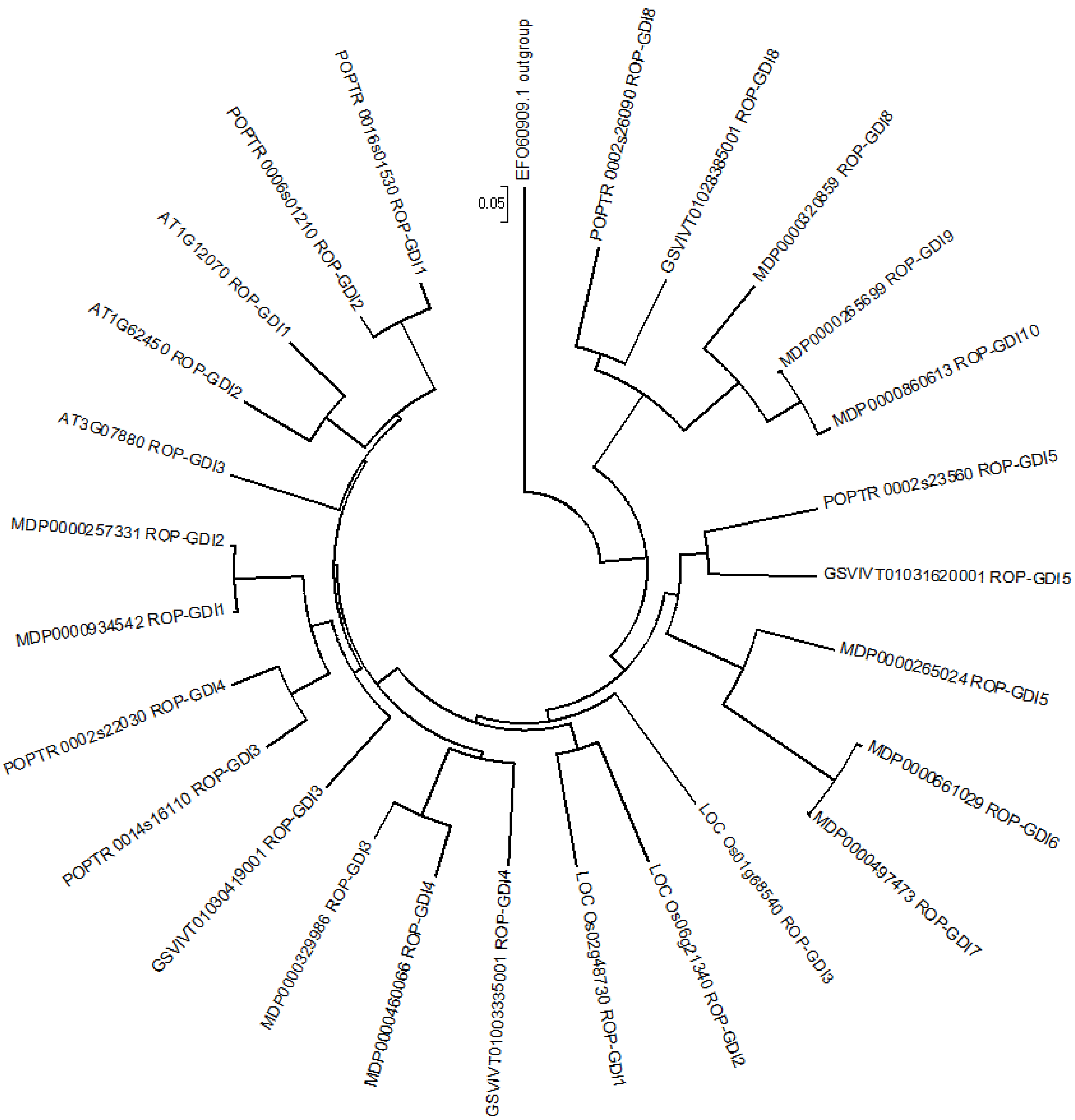

Figure S10. Phenetic tree of ROP-GDI proteins from different plant species including apple. The phenetic tree shows the relationships between the ten ROP-GDI sequences found to be encoded in the *Malus domestica* genome (http://www.rosaceae.org, Velasco *et al.*, 2010) and those from Arabidopsis (*A. thaliana*, identified by AGI number), rice (*O. sativa*, LOC Os), poplar (*P. thricocarpa*, POPTR) and grape (*V. vinifera*, GSVIV) retrieved from the Ensmbl Plants database (http://plants.ensembl.org/index.html). The phenetic tree was constructed by the neighbor-joining method with bootstrapping analysis on the basis of a CLUSTALX alignment (Jeanmougin *et al.*, 1998) and was rooted on the *Giardia lamblia* P15 Rho-GDI protein as outgroup (EFO_60909).

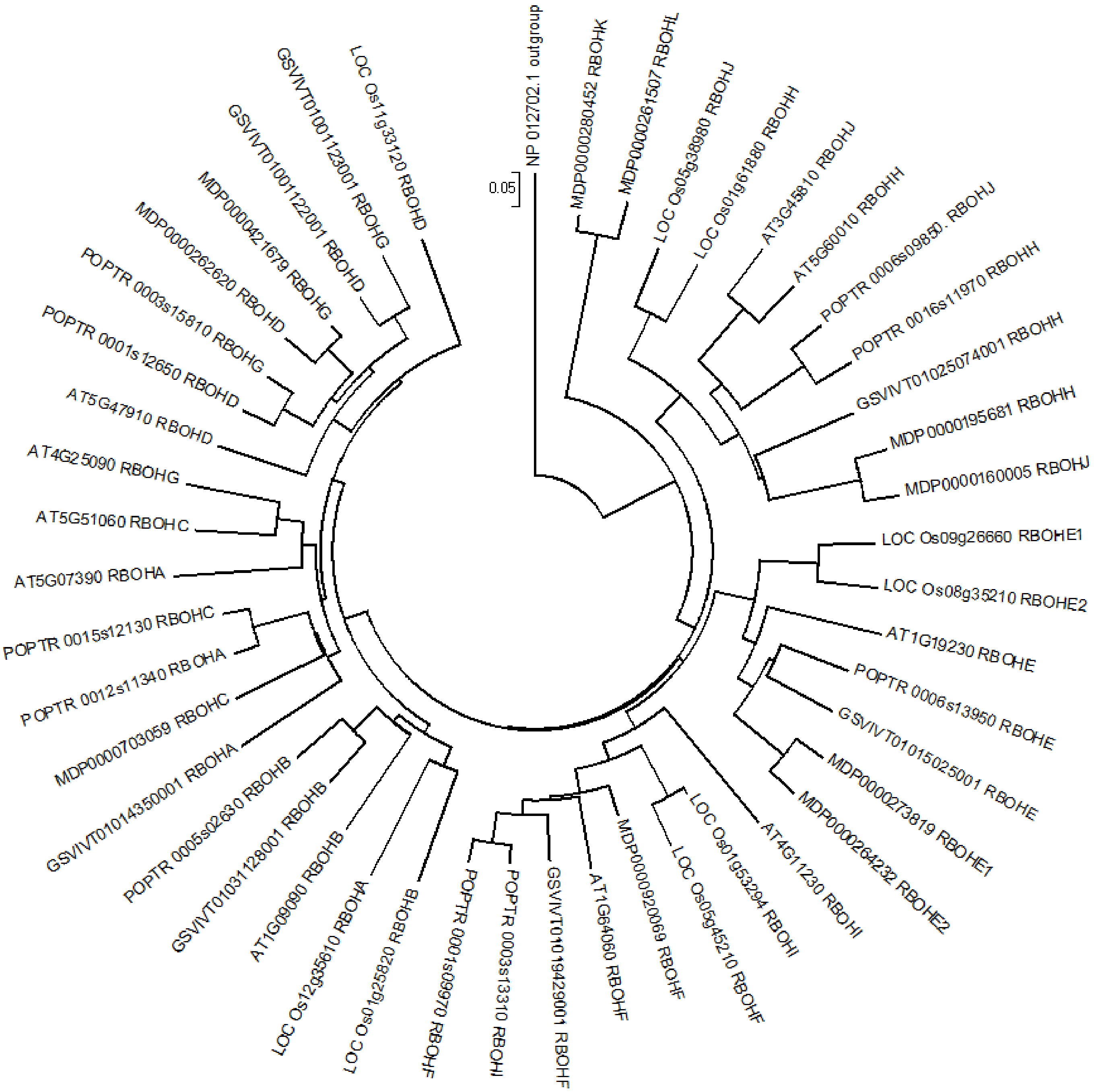

Figure S11. Phenetic tree of RBOH proteins from different plant species including apple. The phenetic tree shows the relationships between the ten RBOH deduced protein sequences found to be encoded in the *Malus domestica* genome (http://www.rosaceae.org, Velasco *et al.*, 2010) and those from Arabidopsis (*A. thaliana*, identified by AGI number), rice (*O. sativa*, LOC Os), poplar (*P. thricocarpa*, POPTR) and grape (*V. vinifera*, GSVIV) retrieved from the Ensmbl Plants database (http://plants.ensembl.org/index.html). Very short apple sequences were excluded from the analysis. The phenetic tree was constructed by the neighbor-joining method with bootstrapping analysis on the basis of a CLUSTALX alignment (Jeanmougin *et al.*, 1998) and was rooted on the *Saccharomices cerevisiae* Fre2p protein as outgroup (NP_012702).

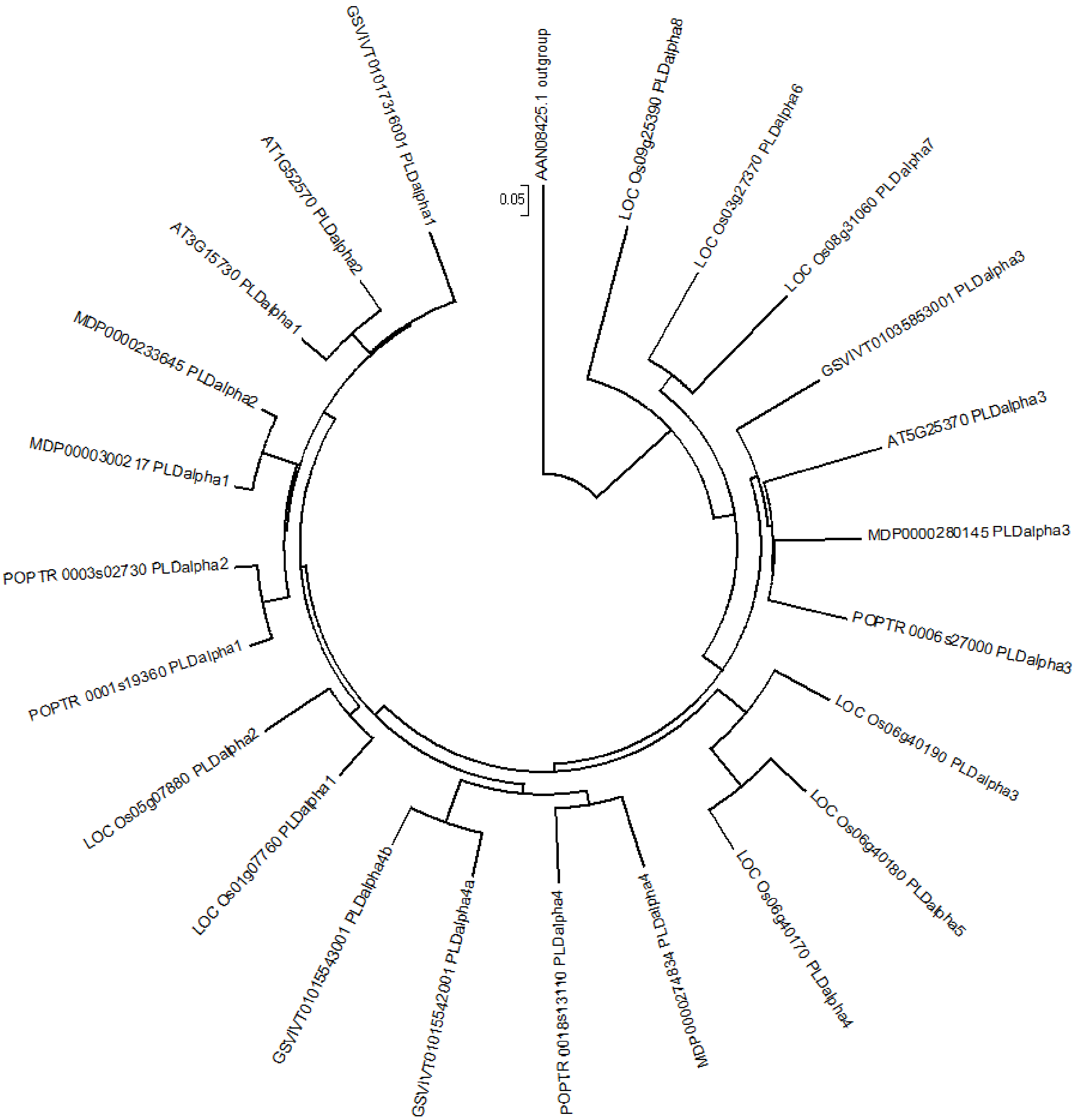

Figure S12. Phenetic tree of PLDα proteins from different plant species including the apple candidates. The phenetic tree shows the relationships among the four identified PLDα sequences found to be encoded in the *Malus domestica* genome (http://www.rosaceae.org, Velasco *et al.*, 2010) and those from Arabidopsis (*A. thaliana*, identified by AGI number), rice (*O. sativa*, LOC Os), poplar (*P. thricocarpa*, POPTR) and grape (*V. vinifera*, GSVIV) retrieved from the Ensmbl Plants database (http://plants.ensembl.org/index.html). The phenetic tree was constructed by the neighbor-joining method with bootstrapping analysis on the basis of a CLUSTALX alignment (Jeanmougin *et al.*, 1998) and was rooted on the *Misgurnus mizolepis* PLDδ1 protein as outgroup (AAN08425).

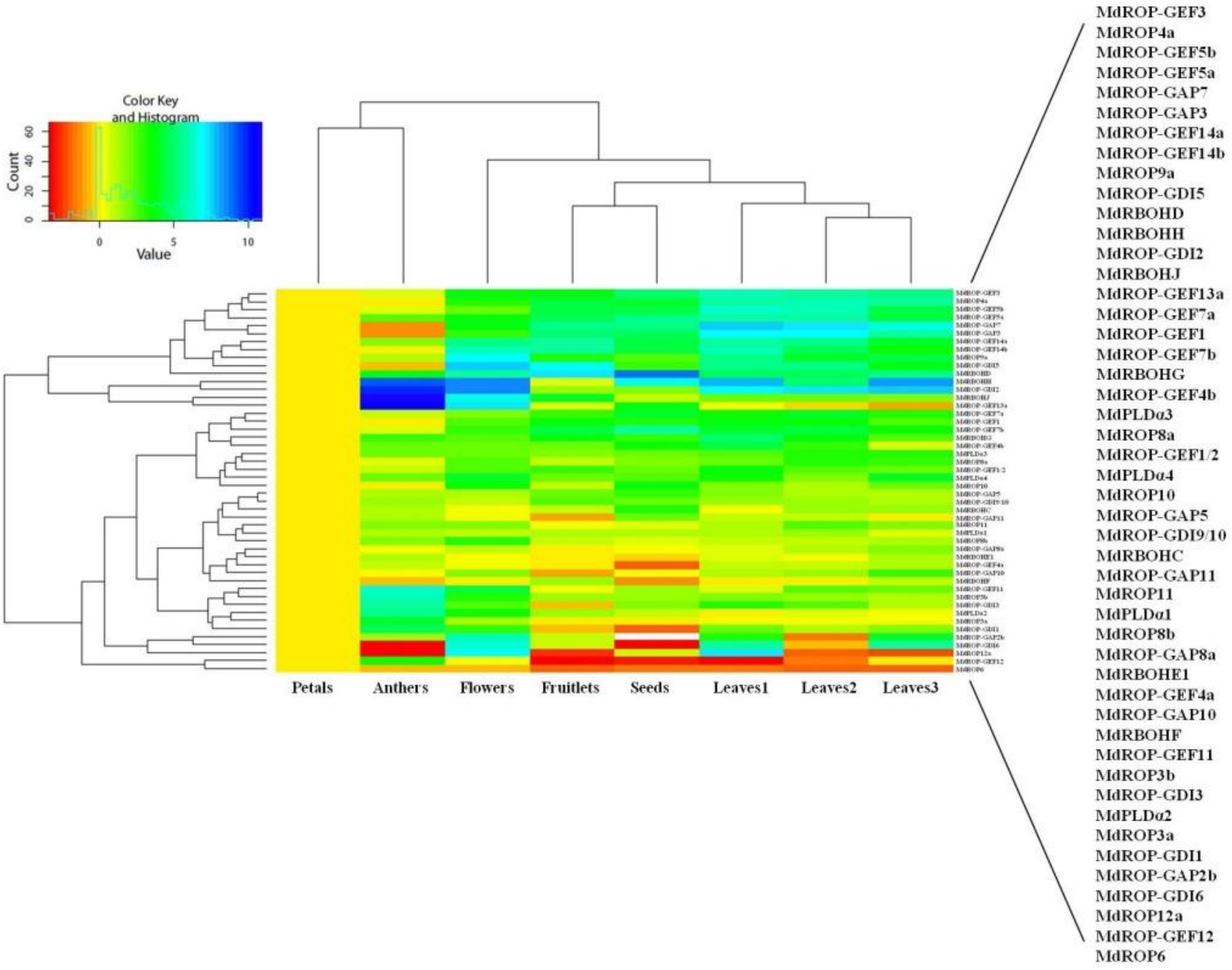

Figure S13. Tissue-specific expression of the apple ROP-GAP rheostat components. The heatmap shows relative tissue-specific expression levels of the components of the ROP-GAP rheostatic machinery identified in apple. Log-transformed expression data obtained by Real-time RT-qPCR on Petals, Anthers, Flowers, Fruitlets, Seeds, and Leaves (at three different stage of growth: 1, 2 and 3 indicate young expanding, fully expanded and mature leaves, respectively) were used for all genes encoding MdROPs, MdROP-GEFs, MdROP-GAPs, MdROP-GDIs, MdRBOHs and MdPLsDα (expanded on the right side of the panel to facilitate reading). Colors ranging from red (down-regulated) to blue (up-regulated) are compared to Petal tissue expression taken as a reference. Yellow indicates no variation of expression. *MdROP3a*, *MdROP3b*, *MdROP-GEF11*, *MdROP-GEF13a*, *MdROP-GDI2*, *MdROP-GDI7*, *MdRBOHH*, *MdRBOHJ* and *MdPLDα2* displayed a significantly higher relative expression level in anthers compared to all other tissues examined.

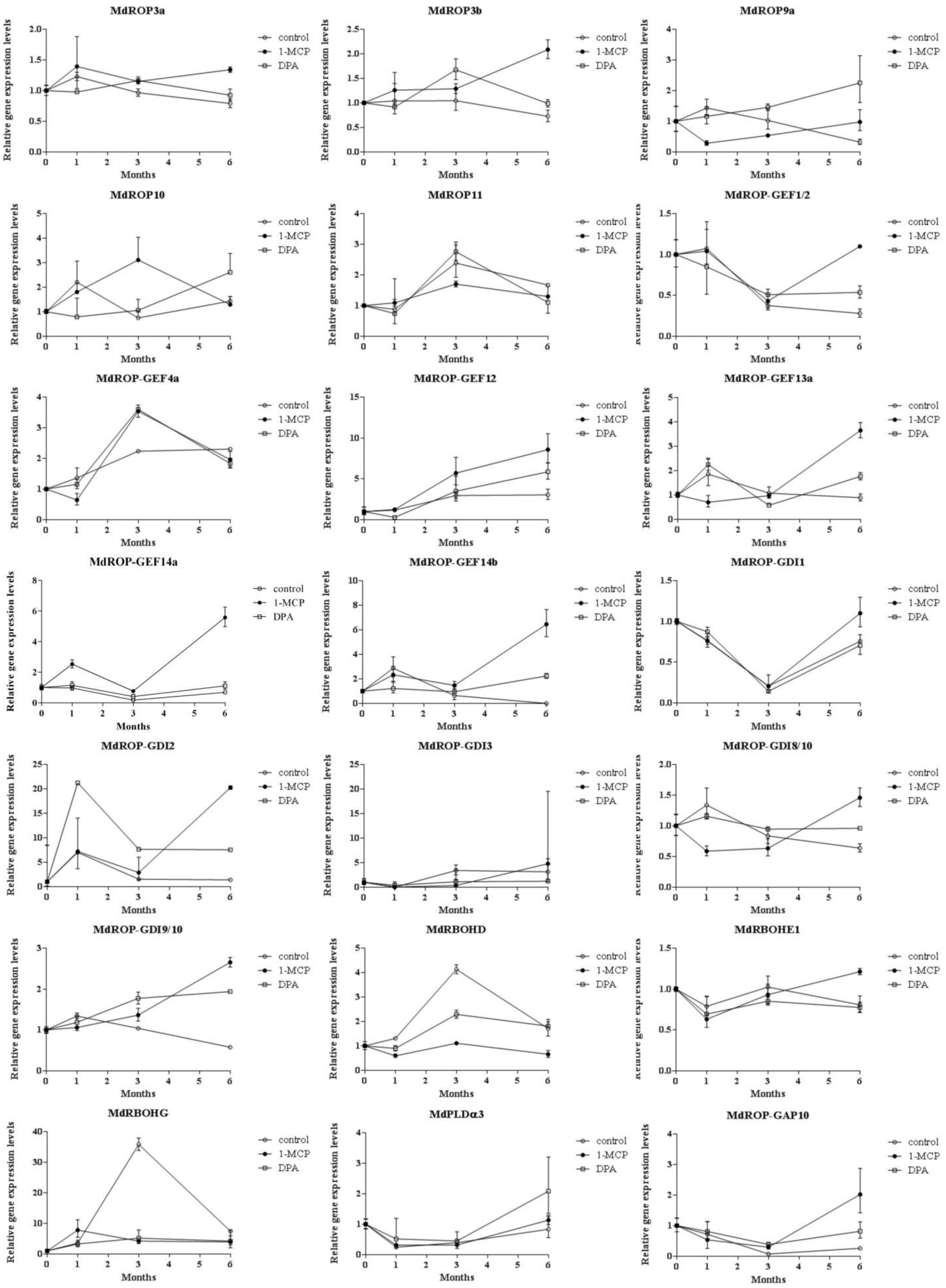
MdROP3a
MdROP3b
MdROP9a
MdROP10
MdROP11
MdROP-GEF1/2
MdROP-GEF4a
MdROP-GEF12
MdROP-GEF13a
MdROP-GEF14a
MdROP-GEF14b
MdROP-GDI1
MdROP-GDI2
MdROP-GDI3
MdROP-GDI8/10
MdROP-GDI9/10
MdRBOHD
MdRBOHE1
MdRBOHG
MdPLDα3
MdROP-GAP10
Relative gene expression levels
Months
control
1-MCP
DPA

Figure S14. Transcriptional expression of the apple ROP-GAP rheostat encoding genes in peels of apple fruits during cold storage. Relative gene expression levels of MdROPs, MdROP-GEFs, MdROP-GAPs, MdROP-GDIs, MdRBOHs and MdPLsDα encoding genes were evaluated by real-time RT-qPCR on RNAs obtained from peel tissues from control, 1-MCP or DPA treated Granny Smith apples at harvest (0) and after 1, 3 and 6 months of cold (1°C) storage in controlled atmosphere (CA)(0.8% O_2, 0.8% CO_2)(harvest 2009/2010). Expression levels were measured relative to Md_8283:1:a (Botton *et al.*, 2011). Each value represents the average of two independent biological replicates ± SD.

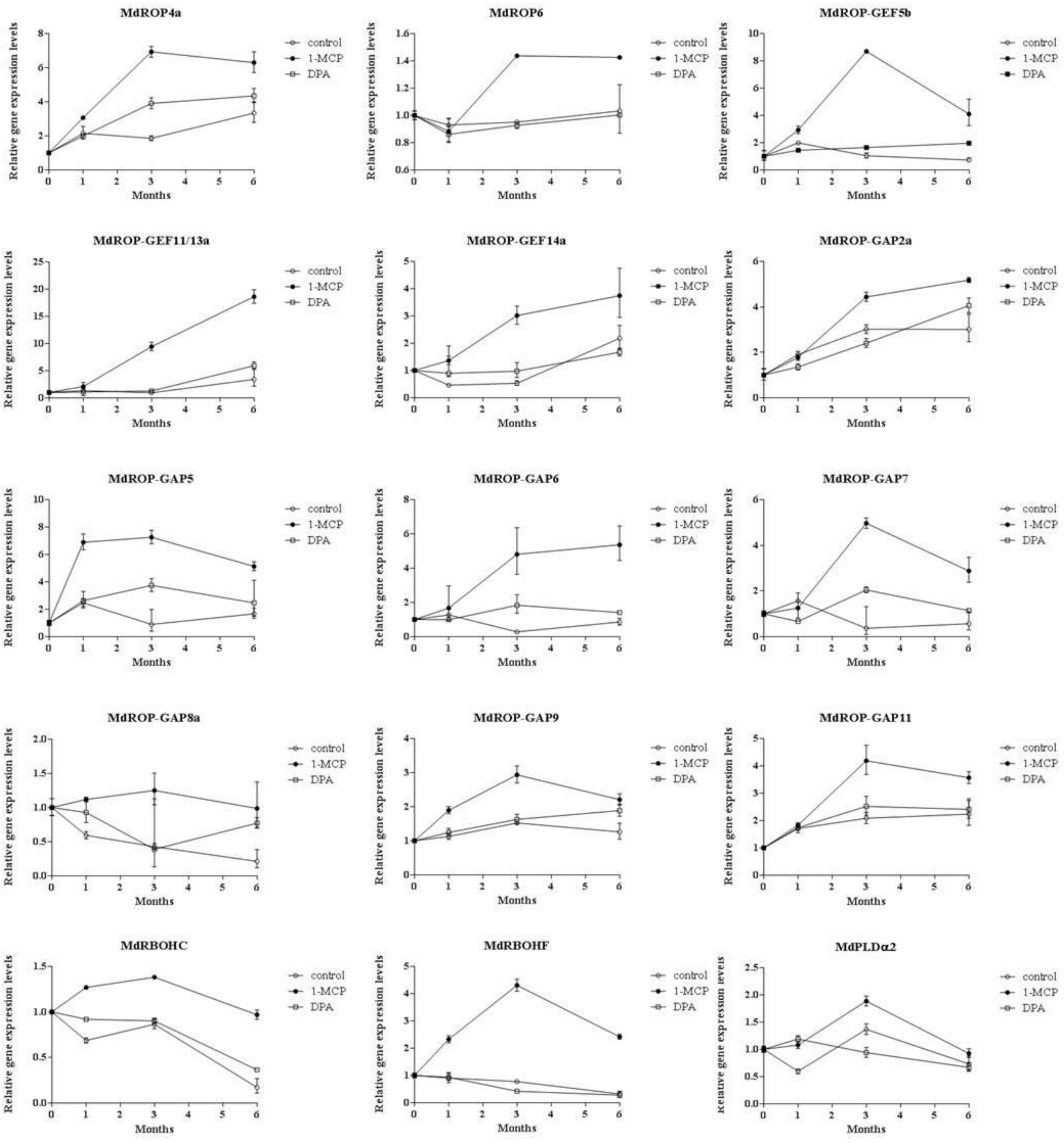

Figure S15. Transcriptional expression of the apple ROP-GAP rheostat encoding genes in peels of apple fruits during cold storage. Relative gene expression levels of MdROPs, MdROP-GEFs, MdROP-GAPs, MdRBOHs and MdPLsDα genes evaluated by real-time RT-qPCR on peels collected from untreated (control)(empty circles), 1-MCP (solid circles) or DPA (empty squares) treated *Granny Smith* apples at harvest (0) and after 1, 3 and 6 months of cold (1°C) storage in controlled atmosphere (CA)(0.8% O_2, 0.8% CO_2)(harvest 2010-2011). Expression levels were

measured relative to Md_8283:1:a (Botton *et al*., 2011). Each value represents the average of two independent biological replicates ± SD.

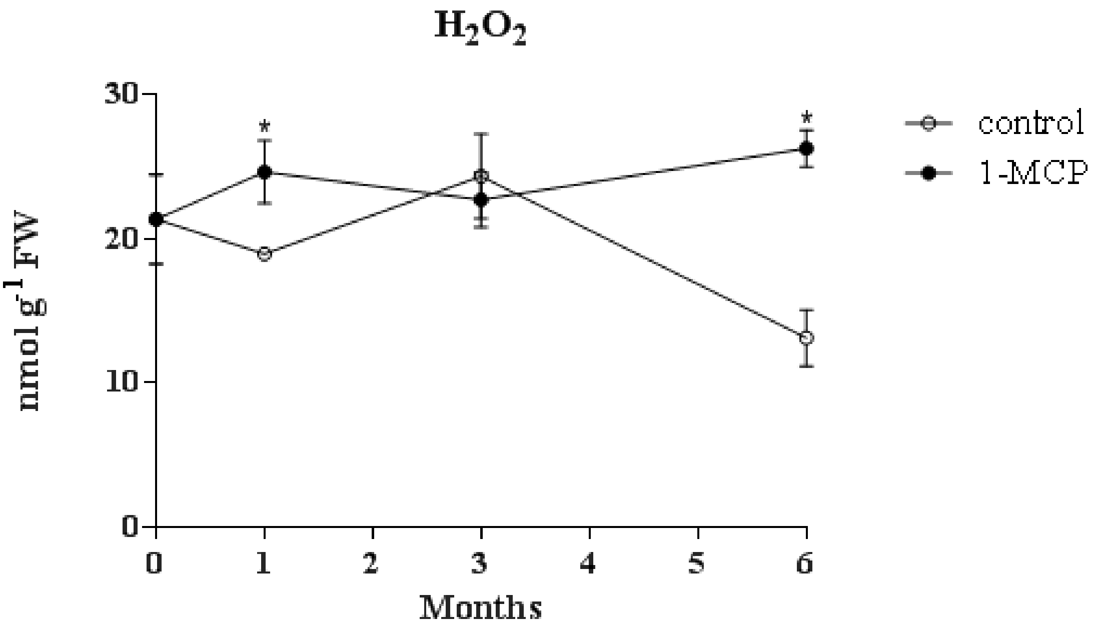

Figure S16. Spectrophotometric determination of H_2O_2 levels in peels of Granny smith apples during cold storage and in response to inhibition of ethylene perception (1-MCP). H_2O_2 levels were quantified spectrophotometrically with the PeroXOquant Quantitative Peroxide Assay kit in peels of untreated control apples (empty circles) or apples treated with 1-MCP (solid circles) at harvest (0) and after 1, 3 and 6 months of cold storage (1°C) in controlled atmosphere (CA, 0.8% O_2, 0.8% CO_2)(harvest 2009/2010). Each value represents the average of three independent replicates ± SD. Asterisks indicate statistically different values (t test, $p<0.05$).

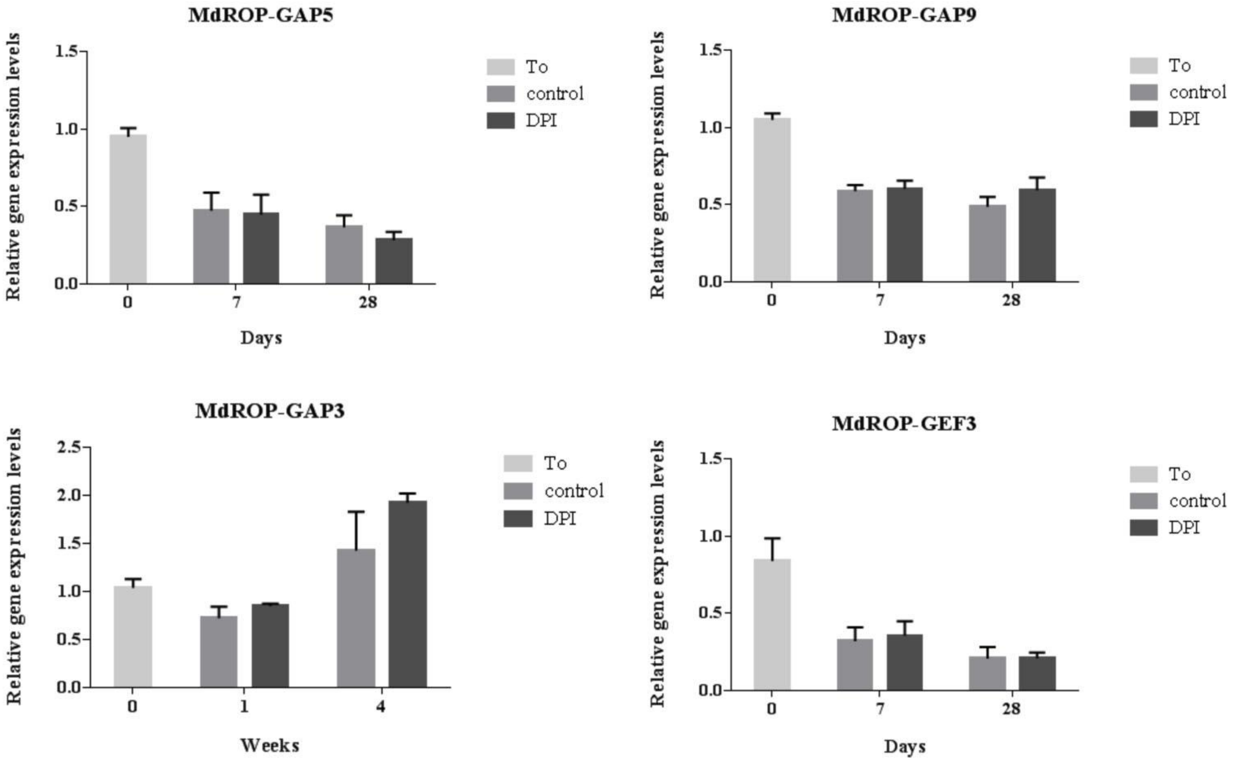

Figure S17. Effects of treatments with 100 µM diphenylene iodonium chloride (DPI) on the expression of the genes encoding ROP-GAP3, ROP-GAP5, ROP-GAP9 and ROP-GEF3 in peels of *Granny smith* apples. Gene expression levels were evaluated by real-time RT-qPCR at harvest (0) and after 1 or 4 weeks of cold storage (1°C) with no treatments (control) or following treatment with 100 µM DPI. Expression levels were measured relative to Md_8283:1:a (Botton et al., 2011). Each value represents the average of three independent biological replicates ± SD.

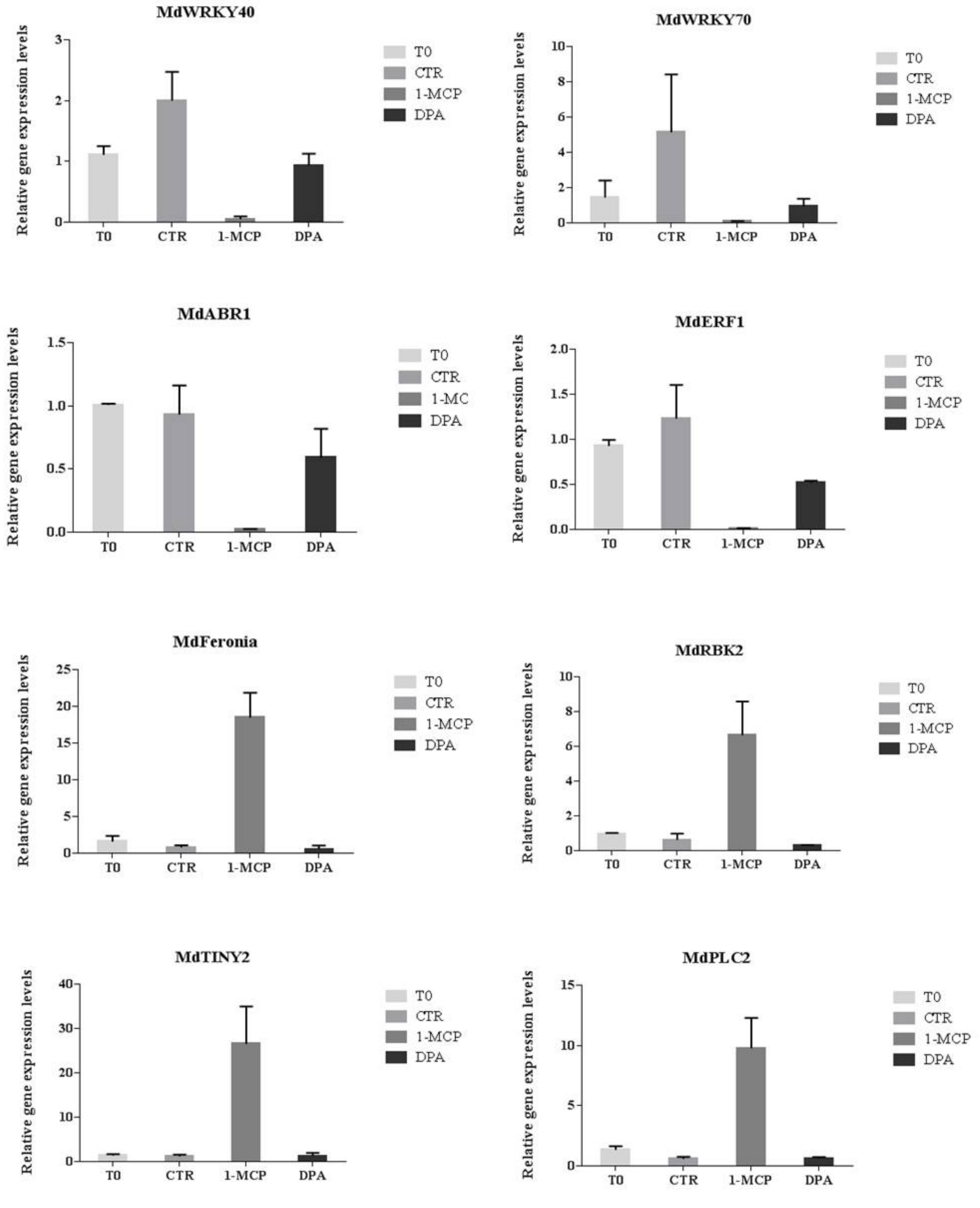

Figure S18 – Effect of cold storage and of 1-MCP or DPA treatments on relative transcript levels of genes involved in ABA and ROP signaling. Differentially expressed genes were selected by

RNA-seq analyses on the base of a five-fold induction/repression filtering threshold (listed in Table 2 and Supplemental Table S18). Relative differential expression was confirmed by real-time qRT-PCR for a subset of genes, including those relevantly related to ABA- (*Feronia*, *WRKY40*, *WRKY70*, *ABR1*, *TINY2*) and ROP-dependent (*Feronia*, *RBK2*) signaling. The relative expression of ERF1 (MDP0000127134) and of *PLC2* (MDP00000239522) is shown as a reference for ethylene and ABA-dependent responses, respectively. Expression levels were evaluated on *Granny Smith* apple peels at harvest (T0) and after 1 month of cold (1°C) storage in controlled atmosphere (CA)(0.8% O2, 0.8% CO2) either in the absence of treatments (CTR) or following treatment with DPA or 1-MCP. Expression levels were measured relative to Md_8283:1:a (Botton *et al.*, 2011). Each value represents the average of three independent biological replicates ± SD.
