# Supplementary material for: Ethylene negatively regulates transcript abundance of ROP-GAP rheostat-encoding genes and affects apoplastic reactive oxygen species homeostasis in epicarps of cold stored apple fruits
Source: J Exp Bot. 2015 Oct 1;66(22):7255–70. doi: 10.1093/jxb/erv422 (PMC4765793; doi:10.1093/jxb/erv422)
Supplement: Supplementary Data [file supp_erv422_jexbot154500_file002.pdf]

Supplementary Tables

Table S1 – Primers pairs used in this work for qRT-PCR experiments. Forward, fw, and reverse, rv, primer pairs are given in 5'-3' orientation. Each primer pair refers to a specific apple sequence (ID), re-named in this work (name) as described in the text, with the exception of MdROP-GEF1/2, MdROP-GDI8/10 and MdROP-GDI9/10 for which the high sequence identity did not permit the design of selective primers pairs. Efficiency of primer pairs was calculated for differentially expressed genes and is shown along with the corresponding r^2. On the last column on the right references are given from which primer pairs were obtained while sequences originally designed in this work are indicated as "this work".

Apple ROP-GAP rheostat genes

ID	*name*	*fw primer*	*rv primer*	*% Efficiency*	*r^2*	*reference*
**MDP0000299673**	MdROP3a	AAAGAAAGGCAAAGGGCAGA	TGTGGGGGAATACATGCAGA			this work
**MDP0000436577**	MdROP3b	AAGGAAGGCGGCAAGGG	TGTGAGGGAGTACACGCAGG			this work
**MDP0000550069**	MdROP4a	AGAAGAGAAAGGGGCAAAGG	CAGAGGAGGTAGCGGTGAAG	112.56	0.981	this work
**MDP0000090045**	MdROP6	CAGTTCAAAAACGCAGCAGA	CGCACTTTCTTCCCAACACT	103.36	0.997	this work
**MDP0000932494**	MdROP8a	CTCTCATTTATACACTTTGTC	TTGCACACTTTCTCTCTGGAA			this work
**MDP0000294582**	MdROP8b	CATTCTTTCCATCCCTCTTTT	AGTACCCAAAACCAATAACCTTA			this work
**MDP0000705111**	MdROP9a	TGCTTTCGTTATGTGGTGTTTT	GCAAACTGCTAAACCTCATCAA			this work
**MDP0000232351**	MdROP10	TCCTTTCGAGTTCCGAGTGT	CAACAGCCTGACGGTGAAG			this work
**MDP0000274576**	MdROP11	CTGGAGTAACCGCGAACATC	TGACCGCTCAAAACAGAAGC			this work
**MDP0000265718**	MdROP12a	TTCCTTGCCAACAATCTTCA	AACTCCCAAAGTCGCATTTTA			this work
**MDP0000306885**	MdROP-GEF1	CCTCTGTGTGTAAAGCGCAGT	CAAAATCAGCCGCGAGTAGTT			this work
**MDP0000306885 MDP0000628931**	MdROP-GEF1/2	AATCTCTCTGCGACGGTGTT	GCCACATTGCCTTTCTCTG			this work
**MDP0000459293**	MdROP-GEF3	ATCACCCAGTCCCTTCTCAA	CGGGGCTTCTACCTATCTGA	113.94	0.982	this work
**MDP0000119721**	MdROP-GEF4a	GTAAGTGTGCGGGAGAAGGA	GGATGTCTGGGACCATGTCT			this work
**MDP0000134252**	MdROP-GEF4b	ATAAGGATGTGGGGCAATCA	GAATGGTTGAACTCGGTTGC			this work
**MDP0000155158**	MdROP-GEF5a	TTGAGGCAAGAAGTGGAAAG	AAACTCCCCCAACTCTCATAA			this work
**MDP0000822948**	MdROP-GEF5b	TTGAGGCAAAATGAGCTTCT	ACTCCCCTAGCTCTCCTGAA	104.7	0.954	this work
**MDP0000922741**	MdROP-GEF7a	GAAACGCTAGGGTCTCACA	GCTGAACACTTCTGCATGGT	96.28	0.954	this work
**MDP0000153185**	MdROP-GEF7b	CGAACACAATGCAAGAAACGA	GCGTGAATGAGTCCTGTGATT			this work
**MDP0000176388 MDP0000238381**	MdROP-GEF11/13a	AGAGAGAGGGGCAGATAGACA	ACTTCCCAATCCTTCAATAGG	116.29	0.961	this work
**MDP0000233239**	MdROP-GEF12	CTGTTGGATTTCATGGGTTG	GTGCCGTTGGACTTCTCAAT			this work
**MDP0000186495**	MdROP-GEF14a	GACGGTGAACAAGGATGAAC	AACATAGAAATGCTGGGGTCT	103.95	0.95	this work
**MDP0000169427**	MdROP-GEF14b	ATTACATTGCTGCCTTGCTG	CCCTACTTGTTCCGCTTTCA			this work
**MDP0000684434**	MdROP-GAP2a	CTTTCTCTGTTTGGCGTTGCT	GCTCCCTCCTGTCACTCTTG			this work
**MDP0000155059**	MdROP-GAP2b	GGAGGAAGGAAGGTTGGAGA	GGCGGTCAGTGAAAACAAAG			this work
**MDP0000463624**	MdROP-GAP3	ACACGGATGAAATGGAGGAG	ACGCTGTAAGCACGAAACCT	105.16	0.977	this work
**MDP0000212513**	MdROP-GAP5	GCTGAGGAATCTGTTTTTGTTCT	GTCACTACCCATTTCACTAGACCT	93.92	0.942	this work
**MDP0000674618**	MdROP-GAP6	GGGAAGCTGAGGAATCTGTT	ACTTAGGCCCACCCATTTTT			this work
**MDP0000237668**	MdROP-GAP7	GGACTCTGGCACATCGTTTT	ATGAGGCTTCCCCATCACTC	99.47	0.958	this work

**MDP0000190245**	MdROP-GAP8a	GCTGATGTTGCACAAATGGA	TGAAATCGAGGTGCTTTTTAAC			this work
**MDP0000279052**	MdROP-GAP9	ATGTATGCCGTCCAAGTGAT	CATCCTCGTCAGAAGGCTCT	122.13	0.993	this work
**MDP0000139755**	MdROP-GAP10	AACAGAAGCTGCGTTATTGGA	AAATCGGGGTACTTTTCAGTTT			this work
**MDP0000163748**	MdROP-GAP11	TGAAGAAAAACTCGACCCAAC	ACCACCCAGCAAGACAGAAA			this work
**MDP0000934542**	MdROP-GDI1	TGGGGAAACTCTTGAACCAG	GCCGGACACAATGTTATTCT			this work
**MDP0000257331**	MdROP-GDI2	GTGGATTTTGAGAATGTTGGA	ACCGGATACGATGTTATTGC			this work
**MDP0000329986**	MdROP-GDI3	CATTGTTTTCCCATGCTGAG	AGAGTTGTGCCAAAGCAAGC			this work
**MDP0000265024**	MdROP-GDI5	AGGGGCTGGTATTGTGTGAG	TTGCTGAAAAACAGATGGAAA			this work
**MDP0000661029**	MdROP-GDI6	GGCTGTCAAAAACTGCTGGA	GCACCCTCACTTCTGGTTCT			this work
**MDP0000320859**<br>**MDP0000860613**	MdROP-GDI8/10	TCGTCAGGTGGGTTTTCATC	TCAGCTTTTCAGCCATTTCC			this work
**MDP0000265699**<br>**MDP0000860613**	MdROP-GDI9/10	GGGCATTTATTCAGCAAAGC	TCACCCCTACAAGGAAAAGC			this work
**MDP0000703059**	MdRBOHC	CGATGCTAGAGTTGGGGTGT	GGGTGGAGGTTTTGTGAGAG	100.06	0.984	this work
**MDP0000262620**	MdRBOHD	GTGGGGGTGTTTTACTGTGG	CTTCGTGGTGGTCTTGTGTG	79.83	0.989	this work
**MDP0000273819**	MdRBOHE1	AAGAGATACCTTTCCGACTTGA	CTTGTCCACCATCAGCAGTG			this work
**MDP0000920069**	MdRBOHF	AAGAACTCAGCCAGCTCTGC	GGAAGGGATATGGATTTGAATG			this work
**MDP0000421679**	MdRBOHG	AGAAACGTGCTCACCAACCT	CGCCGACATACGACTGACT			this work
**MDP0000195681**	MdRBOHH	TTTCGGGTCTCTTGTTTGTTG	GCTCCCTGAGTGTTTTGGTAAG			this work
**MDP0000160005**	MdRBOHJ	CAACTTGGCTACCGCACAT	GGCAGAGCTTCCTGAGTGTT			this work
**MDP0000300217**	MdPLDα1	TGCCAAATCCGACTACCTTC	CCAACCTGCTATGAACATCC	106.92	0.978	this work
**MDP0000233645**	MdPLDα2	GTCGCTTGGGTTGTCAAAGT	GCAGGTTGTGAAGCAGATAAG	112.01	0.979	this work
**MDP0000280145**	MdPLDα3	TGCGGTAAGCAATAATGGAG	AAACACGAGCCTTGGTATCG			this work
**MDP0000274834**	MdPLDα4	GTGGCGAAACCAGTGCTC	CGTCCAACTCTTACATTTTCCA			this work
**MDP0000375455**	Md_8283:1:a	CTCGTCGTCTTGTTCCCTGA	GCCTAAGGACAGGTGGTCTATG			Botton *et al.*, 2011

Ethylene biosynthetic and stress marker genes

GenBank accession number	***name***	***primer fw***	***primer rv***	***reference***
**AB030859**	MdACO1	CAGTCGGATGGGACCAGAA	GCTTGGAATTTCAGGCCAGA	Dal Cin *et al.*, 2005; Botton *et al.*, 2011
**L31347.1**	MdACS	AAGTGGCGAACTGGAGTCGA	GGTTTGATGGGTTCGTGACC	Sabban-Amin *et al.*, 2011
**AY182241.2**	MdAFS	AAGATCCTCAGGCAGCATGG	CTTCACCTTCGAAACCCAGG	Sabban-Amin *et al.*, 2011
**L29450**	MdPPO	CTGACTCGGACTGGTTGGAC	CTTCGCTACTTTGCTCAATGC	this work
**Z48234.1**	MdADH	GGAAGCACTGAAGCCATGAT	CTCCACGACAGAGGGAATGT	this work

RNAseq differentially expressed genes

ID	***name***	***fw primer***	***rv primer***	***% Efficiency***	r^2	***reference***
**MDP0000242979**	MdABR1	TGAGCAGAATCAGCAAATGG	ATGGTGCCTGGAAATCAGAC	96.14	0.986	this work
**MDP0000127134**	MdERF1	ATGGCATAAGGGTGTGGCTA	TAAGCGATTTGCGGACTCTT	106.61	0.997	this work
**MDP0000493959**	MdFERONIA	AGAAGTGCGGTGAAAGGAAC	TGAGAAGATCGTCCCGGATA	126.64	0.951	this work
**MDP00000239522**	MdPLC2	TTCCCATTAACCGTTCCAGA	GCCTCCAAAGTCGTCCTTCT	99.78	0.985	this work
**MDP0000287486**	MdRBK2	TCATGGTTGCTGAACTACGG	GATTGTGCCCTTCTCCAAAA	102.98	0.964	this work
**MDP0000790788**	MdTINY2	TATCATCGTCGTCGTCCTCA	ATACACCCACCCATCCAATG	107.58	0.977	this work
**MDP0000177906**	MdWRKY40	TGAATGGAGCAGTGAATGGA	TTCCCTTGGTTTCTTGAACG	117.40	0.996	this work
**MDP0000175240**	MdWRKY70	CCCAGTGATGTAACGGACAA	GTTGGAAGACACGCAGATCC	105.95	0.993	this work

Table S2 – Tissue specific expression of the genes encoding the apple ROP-GAP machinery. Relative expression of MdROPs, MdROP-GEFs, MdROP-GAPs, MdROP-GDIs, MdRBOHs and MdPLsDα encoding genes (ID) (left panel) was evaluated by real time qRT-PCR in different tissues: Petals, Anthers, (whole) Flowers, (whole) Fruitlets, Seeds, Leaves at three different stage of growth ranging from young expanding (1), fully expanded (2) and mature leaves (3), respectively (central panel) were used. Peel (right panel): apple peels of fruits cv Granny Smith subjected to storage. Petals were considered as reference tissue. Legend: (+) up-regulation/higher relative expression compared to reference; (=) unchanged expression; (-) down-regulation/lower relative expression compared to reference; (×) genes expressed in peels of ripe apple fruits subjected to storage. Absence of symbols indicates that expression could not be detected for a specific gene/tissue combination. MdROP-GAP2a, MdROP-GAP6, MdROP-GAP9, MdROP-GEF11/13a, and MdROP-GDI8/10 were detected only in apple peels, while transcripts of the genes encoding MdRBOHH, MdRBOHJ were not detected in apple peels.

ID	Petals	Anthers	Flowers	Fruitlets	Seed	Leaves1	Leaves2	Leaves3	Peel
**MdROP3a**	Control	+	=	=	=	=	=	=	×
**MdROP3b**	Control	+	+	+	+	+	+	+	×
**MdROP4a**	Control	-	+	+	+	+	+	+	×
**MdROP6**	Control	-	-	-	-	-	-	-	×
**MdROP8a**	Control	+	+	+	+	+	+	+	
**MdROP8b**	Control	+	+	+	+	+	+	+	
**MdROP9a**	Control	+	+	+	+	+	+	+	×
**MdROP10**	Control	=	+	+	+	+	+	+	×
**MdROP11**	Control	+	+	+	+	+	+	+	×
**MdROP12a**	Control	-	+	-	+	+	-	-	
**MdROP-GEF1**	Control	=	+	+	+	+	+	+	×
**MdROP-GEF1/2**	Control	+	+	+	+	+	+	+	×
**MdROP-GEF3**	Control	+	+	+	+	+	+	+	×
**MdROP-GEF4a**	Control	=	+	+	+	+	+	+	×
**MdROP-GEF4b**	Control	+	+	+	+	+	+	+	
**MdROP-GEF5a**	Control	+	+	+	+	+	+	+	
**MdROP-GEF5b**	Control	+	+	+	+	+	+	+	×
**MdROP-GEF7a**	Control	+	+	+	+	+	+	+	×
**MdROP-GEF7b**	Control	+	+	+	+	+	+	+	×
**MdROP-GEF11**	Control	+	+	+	+	+	+	+	
**MdROP-GEF13a**	Control	+	+	+	+	+	-	-	×
**MdROP-GEF11/13a**									×
**MdROP-GEF12**	Control	+	+	-	-	-	-	=	×
**MdROP-GEF14a**	Control	+	+	+	+	+	+	+	×
**MdROP-GEF14b**	Control	=	+	+	+	+	+	+	×
**MdROP-GAP2a**									×
**MdROP-GAP2b**	Control	+	+	+		+	-	+	
**MdROP-GAP3**	Control	-	+	+	+	+	+	+	×
**MdROP-GAP5**	Control	+	+	+	+	+	+	+	×
**MdROP-GAP6**									×

**MdROP-GAP7**	Control	-	+	+	+	+	+	+	×
**MdROP-GAP8a**	Control	+	+	=	+	+	+	+	×
**MdROP-GAP9**	Control								×
**MdROP-GAP10**	Control	+	+	-	+	+	+	+	×
**MdROP-GAP11**	Control	+	+	-	+	+	+	+	×
**MdROP-GDI1**	Control	+	+	-	-	+	+	+	×
**MdROP-GDI2**	Control	+	+	+	+	+	+	+	×
**MdROP-GDI3**	Control	+	+	-	+	+	+	+	×
**MdROP-GDI5**	Control	-	+	+	+	+	+	+	
**MdROP-GDI6**	Control	-	+	+	-	+	-	+	
**MdROP-GDI8/10**									×
**MdROP-GDI9/10**	Control	+	+	+	+	+	+	+	×
**MdRBOHC**	Control	+	+	+	+	+	+	+	×
**MdRBOHD**	Control	+	+	+	+	+	+	+	×
**MdRBOHE1**	Control	+	+	=	-	+	+	+	×
**MdRBOHF**	Control	-	+	+	-	+	+	+	×
**MdRBOHG**	Control	+	+	+	+	+	+	+	×
**MdRBOHH**	Control	+	+	+	+	+	+	+	
**MdRBOHJ**	Control	+	+	+	+	+	+	+	
**MdPLDα1**	Control	+	+	+	+	+	+	+	×
**MdPLDα2**	Control	+	+	+	+	=	+	+	×
**MdPLDα3**	Control	+	+	+	+	+	+	+	×
**MdPLDα4**	Control	+	+	+	+	+	+	+	

Table S3 – Putative ROP encoding sequences indentified in the apple genome. The table shows for each hypothetical ROP sequence: gene ID (gene), number of exons, length (length) of the predicted gene and coding sequence (Cds), chromosome region, strand, corresponding EST(s) previously identified and the proposed name.

GENE	*NUMBER OF EXONS*	*LENGTH*	*CHROMOSOME REGION*	*STRAND*	*EST*	*PROPOSED NAME*
**MDP0000388854**	5	Gene: 1446bp<br>Cds: 408bp	chr2:4564224..4565669	+		MdROP9c
**MDP0000120931**	7	Gene: 1443bp<br>Cds: 606bp	chr2:4554919..4556361	-		MdROP9b
**MDP0000705111**	7	Gene: 1431bp<br>Cds: 603bp	chr15:13122114..13123544	+		MdROP9a
**MDP0000436577**	7	Gene: 2328bp<br>Cds: 594bp	chr2:10853127..10855454	-	GO569695	MdROP3b
**MDP0000299673**	7	Gene: 1833bp<br>Cds: 594bp	chr15:17890018..17891850	-		MdROP3a
**MDP0000932494**	7	Gene: 2552bp<br>Cds: 648bp	chr2:23489373..23491924	-		MdROP8a
**MDP0000294582**	5<br>(truncated)	Gene: 1750bp<br>Cds: 407bp	chr2:23785581..23787330	-		MdROP8b
**MDP0000090045**	7	Gene: 2790bp<br>Cds: 594bp	chr10:7769451..7772240	+	OT041669	MdROP6
**MDP0000425375**	7	Gene:2338 bp<br>Cds: 552 bp	chr8:758290..760627	-		MdROP4b
**MDP0000550069**	7	Gene: 2273bp<br>Cds: 615bp	chr8:747195..749467	-	EB142420	MdROP4a
**MDP0000232351**	8	Gene: 2534bp<br>Cds: 849bp	chr6:20067071..20069604	+	CN857866	MdROP10
**MDP0000274576**	8	Gene: 3064bp<br>Cds: 783bp	chr14:24843517..24846580	-		MdROP11
**MDP0000265718**	4	Gene: 1942bp<br>Cds:1356bp	chr12:519514..521455	+		MdROP12a
**MDP0000269247**	4	Gene: 1942bp<br>Cds:1356bp	chr12:17008838..17010779	-		MdROP12b
**MDP0000853669**	3	Gene:489bp<br>Cds:249bp	chr2:23792960..23793448	-		Absent from phenetic tree (short fragment)

Table S4 – Putative ROP-GEF encoding sequences indentified in the apple genome. The table shows for each hypothetical ROP-GEF sequence: gene ID (gene), number of exons, length (length) of the predicted gene and coding sequence (Cds), chromosome region, strand, corresponding EST(s) previously identified and the proposed name.

GENE	*NUMBER OF EXONS*	*LENGTH*	*CHROMOSOME REGION*	*STRAND*	*EST*	*PROPOSED NAME*
**MDP0000191912**	6	Gene:2390bp<br>Cds: 1593bp	chr6:1566357..1568746	-		MdROP-GEF14c
**MDP0000186495**	6	Gene:2420bp<br>Cds: 1623bp	chr6:1570160..1572579	+		MdROP-GEF14a
**MDP0000922741**	7	Gene:3142bp<br>Cds: 2118bp	chr4:14091884..14095025	-		MdROP-GEF7a
**MDP0000153185**	8	Gene:3160bp<br>Cds: 1989bp	chr12:22516573..22519732	-		MdROP-GEF7b
**MDP0000155158**	7	Gene:4004bp<br>Cds: 1878bp	chr1:18562289..18566292	-		MdROP-GEF5a
**MDP0000822948**	7	Gene:3019bp<br>Cds: 1827bp	chr13:21358198..21361216	+	DR996848	MdROP-GEF5b
**MDP0000119721**	9	Gene:3507bp<br>Cds: 1773bp	unanchored:8472595..8476101	-		MdROP-GEF4a
**MDP0000134252**	7	Gene:2689bp<br>Cds: 1422bp	chr15:37106075..37108763	+	CN888174	MdROP-GEF4b
**MDP0000306885**	8	Gene:4790bp<br>Cds: 2187bp	chr10:13104946..13109735	-	CN884517<br>CN883286<br>CN882640<br>CN855821<br>CN856396<br>CN856512<br>CN857418	MdROP-GEF1
**MDP0000628931**	6	Gene:7759bp<br>Cds: 2055bp	chr9:21337867..21345625	+	CN919478<br>CN919307	MdROP-GEF2
**MDP0000459293**	5	Gene:2822bp<br>Cds: 1655bp	chr8:429923..432744	-		MdROP-GEF3
**MDP0000176388**	7	Gene:2212bp<br>Cds: 1602bp	chr15:11648113..11650324	+	CO052434	MdROP-GEF11
**MDP0000238381**	7	Gene:2250bp<br>Cds: 1605bp	chr2:4047110..4049359	+		MdROP-GEF13a
**MDP0000135163**	7	Gene:2254bp<br>Cds: 1605bp	chr2:4047897..4050150	+		MdROP-GEF13b
**MDP0000233239**	7	Gene:2241bp<br>Cds: 1614bp	chr9:19998242..20000482	-	GO547899<br>GO512660	MdROP-GEF12
**MDP0000169427**	6	Gene:3097bp<br>Cds: 1353bp	chr6:1564135..1567231	+		MdROP-GEF14b
**MDP0000822990**	2	Gene: 778bp<br>Cds: 384bp	chr8:434961..435738	-		Absent from phenetic tree (Short fragment)
**MDP0000490594**	/	Gene: 360bp<br>Cds: 360bp	chr12:20836218..20836577	-		Absent from phenetic tree (Short fragment)
**MDP0000200158**	/	Gene: 360bp<br>Cds: 360bp	chr12:20808431..20808790	-		Absent from phenetic tree (Short fragment)

Table S5 – Putative ROP-GAP encoding sequences indentified in the apple genome. The table shows for each hypothetical ROP-GAP sequence: gene ID (gene), number of exons, length (length) of the predicted gene and coding sequence (Cds), chromosome region, strand, corresponding EST(s) previously identified and the proposed name.

GENE	*NUMBER OF EXONS*	*LENGTH*	*CHROMOSOME REGION*	*STRAND*	*EST*	*PROPOSED NAME*
**MDP0000190245**	5	Gene: 1899bp<br>Cds: 960bp	unanchored:75523280..75525178	-		MdROP-GAP8a
**MDP0000434220**	4	Gene: 1901bp<br>Cds: 951bp	chr12:2575713..2577613	-		MdROP-GAP8b
**MDP0000684434**	4	Gene: 2491bp<br>Cds: 750bp	chr3:14057031..14059521	-		MdROP-GAP2a
**MDP0000139755**	12	Gene: 5823bp<br>Cds: 2331bp	chr14:2029780..2035602	+		MdROP-GAP10
**MDP0000463624**	4	Gene: 2142bp<br>Cds: 1452bp	chr3:31897140..31899281	-		MdROP-GAP3
**MDP0000237668**	4	Gene: 2260bp<br>Cds: 1428bp	chr11:33844483..33846742	+	GO593936<br>GO563182	MdROP-GAP7
**MDP0000155059**	10	Gene:7462 bp<br>Cds: 2088 bp	chr13:24357067..24364528	+		MdROP-GAP2b
**MDP0000163748**	3	Gene:1990 bp<br>Cds: 849 bp	chr3:14059777..14061766	+		MdROP-GAP11
**MDP0000674618**	5	Gene: 3177bp<br>Cds: 1089bp	chr1:11936431..11939607	+		MdROP-GAP6
**MDP0000212513**	5	Gene: 3292bp<br>Cds: 1494bp	chr4:18589894..18593185	-	GO569418<br>CN936392<br>GO568119<br>CN997758	MdROP-GAP5
**MDP0000279052**	29	Gene:12220bp<br>Cds: 4650bp	chr12:2564006..2576229	-	CN911016<br>GO565287	MdROP-GAP9

Table S6 – Putative ROP-GDI encoding sequences indentified in the apple genome. The table shows for each hypothetical ROP-GDI sequence gene ID (gene), number of exons, length (length) of the predicted gene and coding sequence (Cds), chromosome region, strand, corresponding EST(s) previously identified and the proposed name.

GENE	*NUMBER OF EXONS*	*LENGTH*	*CHROMOSOME REGION*	*STRAND*	*EST*	*PROPOSED NAME*
**MDP0000329986**	5	Gene:1741bp<br>Cds: 736bp	chr5:548903..550643	+		MdROP-GDI3
**MDP0000320859**	5	Gene: 2101bp<br>Cds: 840bp	chr10:18100115..18102215	+		MdROP-GDI8
**MDP0000460066**	8	Gene: 4582bp<br>Cds: 1377bp	chr10:32355452..32360033	-		MdROP-GDI4
**MDP0000265024**	5	Gene: 1253bp<br>Cds: 762bp	chr15:3106373..3107625	-	CN912568	MdROP-GDI5
**MDP0000257331**	5	Gene: 2915bp<br>Cds: 687bp	chr17:8884051..8886965	-		MdROP-GDI2
**MDP0000934542**	5	Gene: 2070bp<br>Cds: 687bp	chr9:8258569..8260638	-	GO549025	MdROP-GDI1
**MDP0000661029**	3	Gene: 763bp<br>Cds: 741bp	chr9:801618..802380	+		MdROP-GDI6
**MDP0000265699**	5	Gene: 1695bp<br>Cds: 618bp	chr5:15318145..15319839	+	CO754040	MdROP-GDI9
**MDP0000860613**	5	Gene: 1806bp<br>Cds: 729bp	chr5:15328097..15329902	-	CO754040	MdROP-GDI10
**MDP0000497473**	3	Gene: 763bp<br>Cds: 705bp	chr9:810841..811603	-	CO754040	MdROP-GDI7

Table S7 – Putative RBOH encoding sequences indentified in the apple genome. The table shows for each hypothetical RBOH sequence gene ID (gene), number of exons, length (length) of the predicted gene and coding sequence (Cds), chromosome region, strand, corresponding EST(s) previously identified and the proposed name.

GENE	*NUMBER OF EXONS*	*LENGTH*	*CHROMOSOME REGION*	*STRAND*	*EST*	*PROPOSED NAME*
**MDP0000273819**	17	Gene: 8807 bp<br>Cds: 3195 bp	chr15:15665392..15674198	-	EH009513	MdRBOHE1
**MDP0000264232**	19	Gene: 10910 bp<br>Cds: 3438 bp	chr15:23187659..23198573	-		MdRBOHE2
**MDP0000195681**	14	Gene: 3959 bp<br>Cds: 2661 bp	chr11:9923508..9927466	+		MdRBOHH
**MDP0000160005**	14	Gene: 3975 bp<br>Cds: 2574 bp	chr3:9125577..9129551	-		MdRBOHJ
**MDP0000262620**	11	Gene: 4258 bp<br>Cds: 2898 bp	chr4:6919306..6923563	-		MdRBOHD
**MDP0000703059**	11	Gene: 6479 bp<br>Cds: 2781 bp	chr7:4626933..4633411	+	GO532009<br>CO723039<br>CO541075<br>GO528433	MdRBOHC
**MDP0000920069**	10	Gene: 2760 bp<br>Cds: 1458 bp	chr2:33394241..33397000	+	CN914800<br>GO563304	MdRBOHF
**MDP0000421679**	9	Gene: 3249 bp<br>Cds: 2181 bp	chr8:20040773..20044021	-	CN939426<br>CN948663<br>CV085112	MdRBOHG
**MDP0000280452**	14	Gene :5025 bp<br>Cds: 2451 bp	chr14:26,536,700..26,541,724	-		MdRBOHK
**MDP0000261507**	12	Gene: 5024 bp<br>Cds: 2469 bp	chr14:26,557,660..26,562,683	-		MdRBOHL
**MDP0000302913**	2	Gene: 1327bp<br>Cds: 524bp	unanchored:110512965..110514291	+		Absent from phenetic tree (Short fragment)
**MDP0000303494**	4	Gene: 2637bp<br>Cds: 847bp	chr12:28297616..28300252	-		Absent from phenetic tree (Short fragment)
**MDP0000121332**	4	Gene: 982bp<br>Cds: 705bp	chr9:9249129..9250110	-		Absent from phenetic tree (Short fragment)
**MDP0000289326**	5	Gene: 2003bp<br>Cds:1092bp	chr6:21771774..21773776	+		Absent from phenetic tree (catalytic domain missing)
**MDP0000290071**	6	Gene: 4277bp<br>Cds: 1425bp	chr1:13747012..13751288	+		Absent from phenetic tree (catalytic domain missing)
**MDP0000832599**	6	Gene: 4595bp<br>Cds: 1467bp	chr2:33437666..33442260	+		Absent from phenetic tree (catalytic domain missing)

Table S8 – Putative PLDα encoding sequences indentified in the apple genome. The table shows for each hypothetical PLDα sequence: gene ID (gene), number of exons, length (length) of the predicted gene and coding sequence (Cds), chromosome region, strand, corresponding EST(s) previously identified and the proposed name.

GENE	*NUMBER OF EXONS*	*LENGTH*	*CHROMOSOME REGION*	*STRAND*	*EST*	*PROPOSED NAME*
**MDP0000300217**	4	Gene: 3828bp<br>Cds: 2493 bp	chr6:12278109..12281936	+		MdPLDα1
**MDP0000280145**	3	Gene: 3772bp<br>Cds: 2376 bp	chr15:5997866..6001637	-		MdPLDα3
**MDP0000233645**	6	Gene: 4876bp<br>Cds: 2550bp	chr13:18895431..18900306	+		MdPLDα2
**MDP0000274834**	3	Gene: 5620bp<br>Cds: 2430 bp	chr2:3094311..3099930	-		MdPLDα4

Table S9 - Overview of genes encoding the ROP-GAP rheostat in different plant species including apple. The number of expressed genes encoding ROPs, ROP-GEFs, ROP-GAPs, ROP-GDIs, RBOHs and PLsDα from different plant species (*Vitis vinifera*, *Populus thricocarpa*, *Oryza sativa*) including those identified in this work in the *Malus x domestica* genome are shown**.**

	**ROP**	**ROP-GEF**	**ROP-GAP**	**ROP-GDI**	**RBOH**	**PLDα**
A. thaliana	11	14	6	3	10	3
M. domestica	10	14	10	7	7	4
V. vinifera	8	7	4	4	7	4
P. thricocarpa	12	16	8	6	10	4
O. sativa	6	11	7	3	9	8

Table S10 – Percentage of healthy and superficially scalded fruits (cv *Granny Smith*). The incidence of superficial scald was scored on fruits after six months of storage (harvest 2009/2010) in controlled atmosphere (0.8% O_2, 0.8% CO_2) at 1°C, followed by eight days at room temperature to allow development of scald symptoms. Percentages are referred to as the number of fruits with or without visible scald symptoms over the total number of fruits analyzed on more than 25% of the fruits' surface (control). For DPA fruits with less than 25% of the fruit's surface with scald symptoms were considered in the count due to lower incidence of superficial scald.

	**Healthy fruit**	**Rotten fruit**	**Burns**	**Superficial scald**
**Control**	2.40%	0.30%	0.30%	97.00%
**1-MCP**	94.20%	0.00%	5.50%	0.30%
**DPA**	90.00%	0.40%	2.20%	7.40%

Table S11: Xcel sheet given on a separate file showing RNAseq expression data for the ROP-GAP components in control, 1-MCP or DPA treated apple peels during storage.

Table S12 – Malonydialdehyde (MDA) content in peels of cold stored apples. MDA content (pmol/mg) was determined by HPLC analyses on peels of apples cv *Granny Smith* at harvest (T0) and after 1, 3 and 6 months of cold (1°C) storage in controlled atmosphere (CA, 0.8% O_2 and 0.8% CO_2) without treatments (control) or following treatments with 1-MCP or DPA. sd: standard deviation. Different letters indicate significant differences within the same row (t-test, $p < 0.05$).

	**Control**	**Sd**	**1 MCP**	**sd**	**DPA**	**sd**
**T0**	9.96	1.64	9.96	1.64	9.96	1.64
**1 month**	18.15 **(a)**	2.16	10.08 **(b)**	1.82	26.44 **(a,b)**	11.01
**3 months**	20.55 **(a)**	3.20	8.74 **(b)**	3.83	10.82 **(b)**	2.64
**6 months**	13.20 **(a,b)**	3.96	11.55 **(b)**	1.86	16.17 **(a)**	2.84

Table S13 – HPLC analysis of H_2O_2 content in peels of cold stored apples. H_2O_2 levels were determined by HPLC on peels of apples cv *Granny Smith* at harvest (T0) and after 1, 3 and 6 months of cold (1°C) storage in controlled atmosphere (CA, 0.8% O_2 and 0.8% CO_2) without treatments (control) or following treatments with 1-MCP or DPA. sd: standard deviation. Different letters indicate significant differences within the same row (t-test, $p < 0.05$).

	**Control**	**sd**	**1 MCP**	**sd**	**DPA**	**sd**
**T0**	2.04	0.18	2.04	0.18	2.04	0.18
**1 month**	3.49 **(a)**	0.49	4.46 **(b)**	0.31	3.28 **(a)**	0.25
**3 months**	2.27**(a)**	0.43	2.69**(a)**	0.90	2.27**(a)**	0.44
**6 months**	1.99**(a)**	0.95	3.80**(a)**	1.64	3.14**(a)**	1.10

Table S14 – GSH content in peels of cold stored apples. Total content (nmol/g) of GSH was determined by HPLC in peels of apples cv *Granny Smith* at harvest (T0) and after 1, 3 and 6 months of cold (1°C) storage in controlled atmosphere (CA, 0.8% O_2 and 0.8% CO_2) without treatments (control) or following treatments with 1-MCP or DPA. sd: standard deviation. Different letters indicate significant differences within the same row (t-test, $p < 0.05$).

**GSH**	**Control**	**sd**	**1 MCP**	**sd**	**DPA**	**sd**
**T0**	22.75	10.84	22.75	10.84	22.75	10.84
**1 month**	44.88 **(a)**	17.80	37.33 **(a)**	1.13	24.17 **(a)**	11.47
**3 months**	44.92 **(a,b)**	17.98	36.54 **(a)**	1.05	69.81 **(b)**	9.94
**6 months**	94.66 **(a,b)**	29.89	44.14 **(a)**	4.17	87.35 **(b)**	12.79

Table S15 – Cysteinyl-glycine content in peels of cold stored apples. Cysteinyl-glycine content (nmol/g) was determined by HPLC analysis on peels of apples cv *Granny Smith* at harvest (T0) and after 1, 3 and 6 months of cold (1°C) storage in controlled atmosphere (CA, 0.8% O_2 and 0.8% CO_2) without treatments (control) or following treatments with 1-MCP or DPA. sd: standard deviation. Different letters indicate significant differences within the same row (t-test, $p < 0.05$).

**Cys-gly**	**Control**	**sd**	**1 MCP**	**sd**	**DPA**	**sd**
**T0**	0.01	0.02	0.01	0.02	0.01	0.02
**1 month**	0.08 **(a)**	0.04	0.13 **(a)**	0.04	0.07 **(ab)**	0.05
**3 months**	0.14 **(a)**	0.05	0.07 **(a)**	0.02	0.15 **(ab)**	0.04
**6 months**	0.32 **(c)**	0.09	0.17 **(a)**	0.02	0.15 **(bc)**	0.03

Table S16 – Cysteamine content in peels of cold stored apples. Cysteamine content (nmol/g) was determined by HPLC analysis on peels of apples cv *Granny Smith* at harvest (T0) and after 1, 3 and 6 months of cold (1°C) storage in controlled atmosphere (CA, 0.8% O_2 and 0.8% CO_2) without treatments (control) or following treatments with 1-MCP or DPA. sd: standard deviation. Different letters indicate significant differences within the same row (t-test, $p < 0.05$).

**Cysteamine**	**Control**	**sd**	**1 MCP**	**sd**	**DPA**	**sd**
**T0**	0.57	0.23	0.57	0.23	0.57	0.23
**1 month**	1.55 **(a,b)**	0.24	0.56 **(c)**	0.06	1.22 **(a,c)**	0.22
**3 months**	2.23 **(b,d,e)**	1.41	0.40 **(e)**	0.04	5.51 **(d)**	1.55
**6 months**	2.57 **(b)**	0.82	0.54 **(c)**	0.04	3.71 **(b)**	0.65

Table S17 – Overview of apple "ROS gene network". Sequences were identified by BLAST searches on the *Rosaceae* database (www.rosaceae.org) using the *A. thaliana* sequences (described by Mittler *et al.*, 2011) as queries. The apple "ROS gene network" is composed of 316 genes encoding 30 superoxide dismutases (SOD), 26 ascorbate peroxidases (APXs), 8 monodehydroascorbate reductases (MDHARs), 10 dehydroascorbate reductases (DHARs), 3 glutathione reductases (GRs), 5 catalases (CATs), 19 glutathione peroxidases (GPXs), 14 ferritins, 41 hypothetical blue copper proteins, 20 NADPH oxidase-like proteins, 8 alternative oxidases (AOXs), 18 peroxiredoxins (PrxRs), 66 hypothetical thioredoxins (TRXs) and 48 putative glutaredoxins (GLRs).

Gene family	*Arabidopsis thaliana*	*Malus domestica*
Superoxide Dismutase (SOD)	At4g25100.3	/
$O_2^- + O_2^- + 2H^+ \rightarrow H_2O_2 + O_2$	At5g51100.1	MDP0000294567
		MDP0000181188
		MDP0000243650
		MDP0000127652
		MDP0000123488
	At5g23310.1	MDP0000374181
		MDP0000272757
		MDP0000162292
		MDP0000222804
		MDP0000187560
	At1g08830.1	MDP0000662094
		MDP0000272510
		MDP0000201158
		MDP0000121919
		MDP0000321336
		MDP0000188546
		MDP0000489706
	At2g28190.1	MDP0000250286
		MDP0000318172
		MDP0000258717
	At5g18100.1	MDP0000364366
		MDP0000315650
	At3g10920.1	MDP0000220086
		MDP0000278922
		MDP0000688410
		MDP0000281277
		MDP0000138103

		MDP0000387371
		MDP0000187714
	At3g56350.1	MDP0000173023
Ascorbate Peroxidase (APX)	At1g07890.1	MDP0000241173
2 Asc + H_2O_2 → 2 MDA + $2H_2O$		MDP0000254826
		MDP0000261341
		MDP0000199034
	At3g09640.1	MDP0000126107
		MDP0000192572
		MDP0000210077
		MDP0000701945
		MDP0000399965
	At4g35000.1	MDP0000189320
		MDP0000234905
		MDP0000021998
		MDP0000316890
		MDP0000151342
		MDP0000214851
		MDP0000282062
		MDP0000169497
		MDP0000793434
	At4g09010.1	/
	At4g35970.1	/
	At4g32320.1	MDP0000943804
		MDP0000143123
		MDP0000903820
	At1g33660.1	/
	At4g08390.2	/
	At1g77490.1	MDP0000897274
		MDP0000918790
		MDP0000483271
		MDP0000248823
		MDP0000207771
Monodehydroascorbate Reductase (MDHAR)	At1g63940.4	/
MDHA + NAD(P)H + H^+ → Asc + $NAD(P)^-$	At3g09940.1	/
	At3g27820.1	MDP0000320539
		MDP0000164300
		MDP0000152184
	At3g52880.1	MDP0000140206

		MDP0000157871
		MDP0000267350
		MDP0000261821
		MDP0000199989
	At5g03630.1	/
Dehydroascorbate Reductase (DHAR)	At5g16710.1	MDP0000530903
DHA + 2 GSH → Asc + GSSG		MDP0000240690
		MDP0000175246
		MDP0000156763
	At5g36270.1	/
	At1g75270.1	MDP0000127419
		MDP0000311865
		MDP0000316839
		MDP0000146156
		MDP0000942136
	At1g19550.1	/
	At1g19570.1	MDP0000236168
Glutathione Reductase (GR)	At3g24170.1	MDP0000300208
GSSG + NAD(P)H → 2 GSH + $NAD(P)^-$		MDP0000576268
	At3g54660.1	MDP0000202123
Catalase (Cat)	At1g20630.1	/
$2H_2O_2 \rightarrow 2H_2O + O_2$	At4g35090.1	MDP0000132452
		MDP0000147628
		MDP0000699607
		MDP0000678891
		MDP0000309331
	At1g20620.1	/
Glutathione Peroxidase (GPX)	At2g25080.1	/
H_2O_2 + 2 GSH → $2H_2O$ + GSSG	At2g31570.1	MDP0000251176
		MDP0000291593
	At2g43350.1	MDP0000264931
		MDP0000282034
	At2g48150.1	MDP0000647547
		MDP0000243057
	At3g63080.1	MDP0000751256
	At4g31870.1	MDP0000311291
	At1g63460.1	MDP0000212661
		MDP0000338065
		MDP0000203927

		MDP0000191008
		MDP0000258603
		MDP0000207137
		MDP0000302772
	At4g11600.1	MDP0000365920
		MDP0000913598
		MDP0000180721
		MDP0000243843
Ferritin	At5g01600.1	MDP0000189389
Fe + P → P-Fe		MDP0000119928
		MDP0000286750
		MDP0000317816
	At3g56090.1	/
	At2g40300.1	MDP0000152866
		MDP0000385350
		MDP0000325832
		MDP0000252706
		MDP0000870126
	At3g11050.1	MDP0000262639
		MDP0000212807
		MDP0000230140
		MDP0000229741
		MDP0000140963
Blue copper protein	At5g20230.1	MDP0000808076
Cu + P → P-Cu		MDP0000470916
		MDP0000507001
		MDP0000375032
		MDP0000866270
		MDP0000118766
		MDP0000619261
		MDP0000286604
		MDP0000479478
		MDP0000744832
		MDP0000264592
		MDP0000164201
		MDP0000933335
		MDP0000209523
		MDP0000299980
		MDP0000213863
		MDP0000610447
		MDP0000269284

		MDP0000204569
		MDP0000129648
		MDP0000569069
		MDP0000206710
		MDP0000248730
	At1g72230.1	MDP0000873376
		MDP0000163314
	At3g27200.1	MDP0000284556
		MDP0000258325
		MDP0000202045
		MDP0000208735
		MDP0000231401
		MDP0000588940
	At3g60280.1	MDP0000162466
	At4g12880.1	/
	At5g26330.1	MDP0000181736
		MDP0000669625
		MDP0000830099
		MDP0000286477
		MDP0000142111
		MDP0000589057
	At2g33740.1	MDP0000231967
		MDP0000186074
	At4g28365.1	MDP0000161510
	At2g31050.1	/
NADPH oxidase-like	At5g23980.1	MDP0000742438
NADPH + e^- + O_2 → $NADP^-$ + O_2^- + H^+ (?)	At5g49730.1	/
	At5g50160.1	MDP0000303779
		MDP0000272115
	At1g01580.1	MDP0000214984
		MDP0000226559
		MDP0000225549
		MDP0000144724
	At5g49740.1	MDP0000151434
		MDP0000138686
		MDP0000299273
		MDP0000287362
	At5g23990.1	MDP0000613837
	At1g01590.1	/
	At5g67590.1	MDP0000330039

		MDP0000259855
		MDP0000330038
		MDP0000202050
		MDP0000272802
		MDP0000682771
		MDP0000151721
		MDP0000258055
	At1g23020.1	/
Alternative Oxidase (AOX)	At1g32350.1	/
$2e^{-} + 2H^{+} + O_2 \rightarrow H_2O$	At3g22370.1	/
	At3g22360.1	MDP0000940411
	At3g27620.1	/
	At5g64210.1	MDP0000643331
		MDP0000874020
		MDP0000323076
		MDP0000244591
	At4g22260.1	MDP0000200740
		MDP0000195881
		MDP0000131372
Peroxiredoxin (PrxR)	At1g48130.1	MDP0000159365
$2P\text{-}SH + H_2O_2 \rightarrow P\text{-}S\text{-}S\text{-}P + 2H_2O$		MDP0000232332
	At3g11630.1	/
	At5g06290.1	MDP0000200810
		MDP0000633462
		MDP0000320612
	At3g06050.1	MDP0000258515
		MDP0000884441
	At3g26060.1	MDP0000247659
		MDP0000755936
	At1g65990.1	/
	At1g65980.1	MDP0000519575
		MDP0000293090
		MDP0000244884
		MDP0000705110
	At1g65970.1	/
	At1g60740.1	/
	At3g52960.1	MDP0000673491
		MDP0000383765
		MDP0000148952
		MDP0000614959
		MDP0000188780

	At3g03405.1	/
Thioredoxins (TRX)	At2g04700.1	MDP0000203322
P-S-S-P + $2H^+ \rightarrow$ 2P-SH		MDP0000252195
	At1g62180.1	MDP0000279311
		MDP0000167383
	At1g43560.1	/
	At1g31020.1	/
	At1g52990.1	/
	At1g53300.1	MDP0000226590
		MDP0000553412
		MDP0000501387
	At1g76760.1	MDP0000167378
		MDP0000419574
		MDP0000261677
		MDP0000273793
	At2g33270.1	/
	At2g42580.1	MDP0000146444
		MDP0000252130
	At3g06730.1	MDP0000863789
	At3g08710.1	MDP0000235775
		MDP0000597542
		MDP0000316074
		MDP0000390676
		MDP0000445373
		MDP0000249115
		MDP0000613487
	At3g20560.1	MDP0000143707
		MDP0000206232
		MDP0000230558
	At3g56420.1	/
	At4g04950.1	MDP0000302965
		MDP0000898292
		MDP0000397437
	At3g56420.1	/
	At4g29670.2	MDP0000279178
		MDP0000595987
		MDP0000138960
		MDP0000209701
	At4g32580.1	/
	At4g37200.1	MDP0000279928
		MDP0000680639

		MDP0000196226
		MDP0000321876
	At2g40790.1	/
	At3g51030.1	MDP0000415439
		MDP0000622392
		MDP0000166089
	At5g39950.1	MDP0000448333
		MDP0000546099
		MDP0000132688
		MDP000069018
		MDP0000391509
		MDP0000752795
	At5g42980.1	/
	At1g19730.1	MDP0000322266
		MDP0000562983
	At1g45145.1	/
	At1g03680.1	/
	At4g03520.1	MDP0000794149
		MDP0000686419
	At2g15570.1	MDP0000251669
	At3g15360.1	MDP0000626628
		MDP0000323884
		MDP0000194903
	At4g35460.1	/
	At2g17420.1	MDP0000251344
		MDP0000235846
		MDP0000823251
		MDP0000233802
	At2g41680.1	MDP0000525742
		MDP0000845788
		MDP0000314335
		MDP0000394567
	At1g50320.1	MDP0000268156
		MDP0000926084
		MDP0000431533
		MDP0000481941
		MDP0000290274
		MDP0000670699
Glutaredoxin (GLR)	At1g03850.2	/
DHA + 2 GSH → Asc + GSSG	At1g06830.1	/
	At1g28480.1	MDP0000713715

	MDP0000135807
	MDP0000179654
At2g20270.1	/
At2g30540.1	MDP0000724699
	MDP0000757379
	MDP0000804081
At2g47870.1	MDP0000870722
	MDP0000768644
At2g47880.1	/
At3g02000.1	MDP0000148389
	MDP0000752328
	MDP0000156398
At3g62930.1	MDP0000295074
	MDP0000155448
	MDP0000262876
At3g62950.1	MDP0000244038
	MDP0000406592
	MDP0000272528
	MDP0000804078
	MDP0000725469
At3g62960.1	/
At4g15660.1	/
At4g15660.1	/
At4g15680.1	/
At4g15690.1	/
At4g15700.1	/
At4g28730.1	MDP0000341029
At4g33040.1	MDP0000341028
	MDP0000934046
	MDP0000906801
	MDP0000579840
	MDP0000330164
At5g11930.1	/
At5g14070.1	MDP0000781442
	MDP0000432422
	MDP0000177956
	MDP0000788727
	MDP0000823096
At5g18600.1	MDP0000892318
	MDP0000376239
	MDP0000472203

	At1g77370.1	MDP0000216436
		MDP0000456271
	At5g20500.1	MDP0000850178
	At5g40370.1	MDP0000642077
		MDP0000284842
		MDP0000243764
	At5g63030.1	MDP0000144735
	At3g11920.1	MDP0000284515
		MDP0000277069
		MDP0000183490
	At4g10630	MDP0000249252
		MDP0000206005

Supplemental Table S18

Xcel sheet given on a separate file showing genes related to ROP signaling and differentially expressed between 1-MCP and control apple skins after 1 month of cold storage

Supplemental Table S19

Xcel sheet given on a separate file showing GO enrichment analysis of *MdRBOHC* co-expressed genes
